# Single‐Cell RNA Sequencing of Human Lung Tissues Reveals Metallothionein‐Positive T Cells as a Novel Potential Marker of Susceptibility to Chronic Obstructive Pulmonary Disease

**DOI:** 10.1002/advs.202509332

**Published:** 2026-02-13

**Authors:** Zengqing Liu, Xiaoxia Ren, Mi Mu, Huanyu Long, Jialu Lv, Zhihong Feng, Jie Liu, Jiajia Wang, Hui Deng, Fanyu Shi, Muzhi Zhang, Ruoyang Zhang, Yang Liu, Mengyu Xu, Yang Fan, Zengtao Wang, Zhe Lv, Yan Chen, Chris J. Corrigan, Ying Sun, Yahong Chen, Chen Chen, Ye Cui, Wei Wang

**Affiliations:** ^1^ Department of Immunology School of Basic Medical Sciences Capital Medical University Beijing China; ^2^ Department of Respiratory Medicine Capital Medical University Beijing China; ^3^ Immune Dysfunction and Pulmonary Fibrosis Joint Laboratory For Clinical Medicine Capital Medical University Beijing China; ^4^ Department of Pulmonary and Critical Care Medicine Center of Respiratory Medicine China‐Japan Friendship Hospital Beijing China; ^5^ Department of Respiratory and Critical Care Medicine The Eighth Medical Center of Chinese PLA General Hospital Beijing China; ^6^ Department of Pulmonary and Critical Care Medicine Peking University Third Hospital Beijing China; ^7^ Department of Respiratory Medicine Beijing Xuanwu Hospital Capital Medical University Beijing China; ^8^ Biomedical Innovation Center Beijing Shijitan Hospital Capital Medical University Beijing China; ^9^ Faculty of Life Sciences & Medicine School of Immunology & Microbial Sciences King's College London London UK

**Keywords:** biomarker, chronic obstructive pulmonary disease, single‐cell RNA sequence analysis, T Cells

## Abstract

Chronic obstructive pulmonary disease (COPD) is a highly heterogeneous disease with complex pathogenesis. Identifying high‐risk populations and implementing timely prevention strategies are critical to reducing the disease burden. Single‐cell RNA sequencing of lung tissue from control never‐smokers, patients with pre‐COPD, and COPD patients revealed a novel T cell subset characterized by high expression of metallothionein (MT) genes, designated MT‐high T cells. These cells were progressively depleted in the lungs with disease progression. A similar decline was observed in the peripheral blood using flow cytometry, highlighting the potential of these cells to serve as an accessible biomarker of disease progression. Functional analysis indicated that MT‐high T cells suppress CD8^+^ T cell cytotoxic activity, suggesting a key immunoregulatory role in disease pathogenesis. Receiver operating characteristic curve analysis demonstrated the excellent potential of MT‐high T cell frequency to predict susceptibility to COPD. These findings establish MT‐high T cells as promising biomarkers for identifying individuals at risk for COPD and as novel targets for future therapeutic and prophylactic strategies.

## Graphical abstract

Using single‐cell RNA sequencing of lung samples and flow cytometry of peripheral blood, we identified a novel T cell subset with high metallothionein (MT) expression. These MT‐high T cells suppress CD8^+^ T cell cytotoxicity and are progressively depleted from healthy controls to chronic obstructive pulmonary disease (COPD) patients, positioning them as early biomarkers for COPD risk and key regulators of disease pathogenesis.

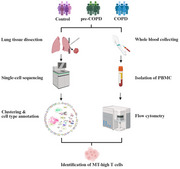

## 1 Introduction

Chronic obstructive pulmonary disease (COPD) is a leading cause of morbidity and mortality worldwide, characterized by persistent respiratory symptoms and progressive airflow limitation. It is a major public health concern, with an estimated 392 million individuals affected globally and at least 3.3 million deaths annually, according to the Global Burden of Disease (GBD) study [1, 2]. COPD is typically diagnosed using spirometry, with a forced expiratory volume in 1 s (FEV1) to forced vital capacity (FVC) ratio of less than 0.7 considered the gold standard for diagnosis [3]. Reliance solely on spirometric criteria for diagnosis does, however, have considerable limitations, most obviously and critically the fact that early changes in the airways of patients liable to develop COPD may not result in measurable airflow obstruction, leading frequently to a missed diagnosis of the disease in its early stages when intervention is likely to be most effective [4, 5, 6]. In addition, the irreversible pathological changes in the airways seen in later stages of the disease are often very advanced before the patient takes heed of the symptoms and seeks medical advice, a diagnostic dilemma further deepened by the lack of any known biomarkers of disease susceptibility [7]. Thus, there is a critical need for early identification of at‐risk individuals and the development of targeted preventive strategies to slow or halt disease progression.

Recent advances in genome sequencing technologies have significantly enhanced our understanding of the molecular underpinning of respiratory diseases, including COPD [8]. Single‐cell RNA sequencing (scRNA‐seq) has emerged as a powerful tool, offering unparalleled resolution and enabling detailed exploration of cellular diversity within tissues. ScRNA‐seq studies have increasingly focused on the heterogeneity of various lung cell populations, including alveolar and airway epithelial cells [9, 10, 11, 12, 13, 14, 15], stromal cells [16], and immune cells [17, 18, 19]. These studies have significantly advanced our understanding of the cellular and molecular processes driving COPD. In the present study, we employed scRNA‐seq to investigate the cellular landscape of lung tissues from healthy individuals, pre‐COPD patients and stable‐stage COPD patients, focusing on transcriptional profiles of airway immune cells to characterize the altered immune landscape in COPD, particularly during the early stage of the disease. Our analysis has identified a previously unreported T cell subpopulation which we have termed “metallothionein (MT)‐high T cells” owing to their elevated metallothionein expression. These cells are detectable in peripheral blood, and their declining numbers serve as a marker of the risk of developing COPD prior to disease establishment, potentially enabling future prophylactic therapy and disease prevention. Additionally, we have investigated the potential immune modulatory activity of these cells relevant to their roles in arresting of the development and progression of COPD.

## 2 Results

### 2.1 Lung Cell Atlas of COPD Patients and Control Donors

**FIGURE 1:**
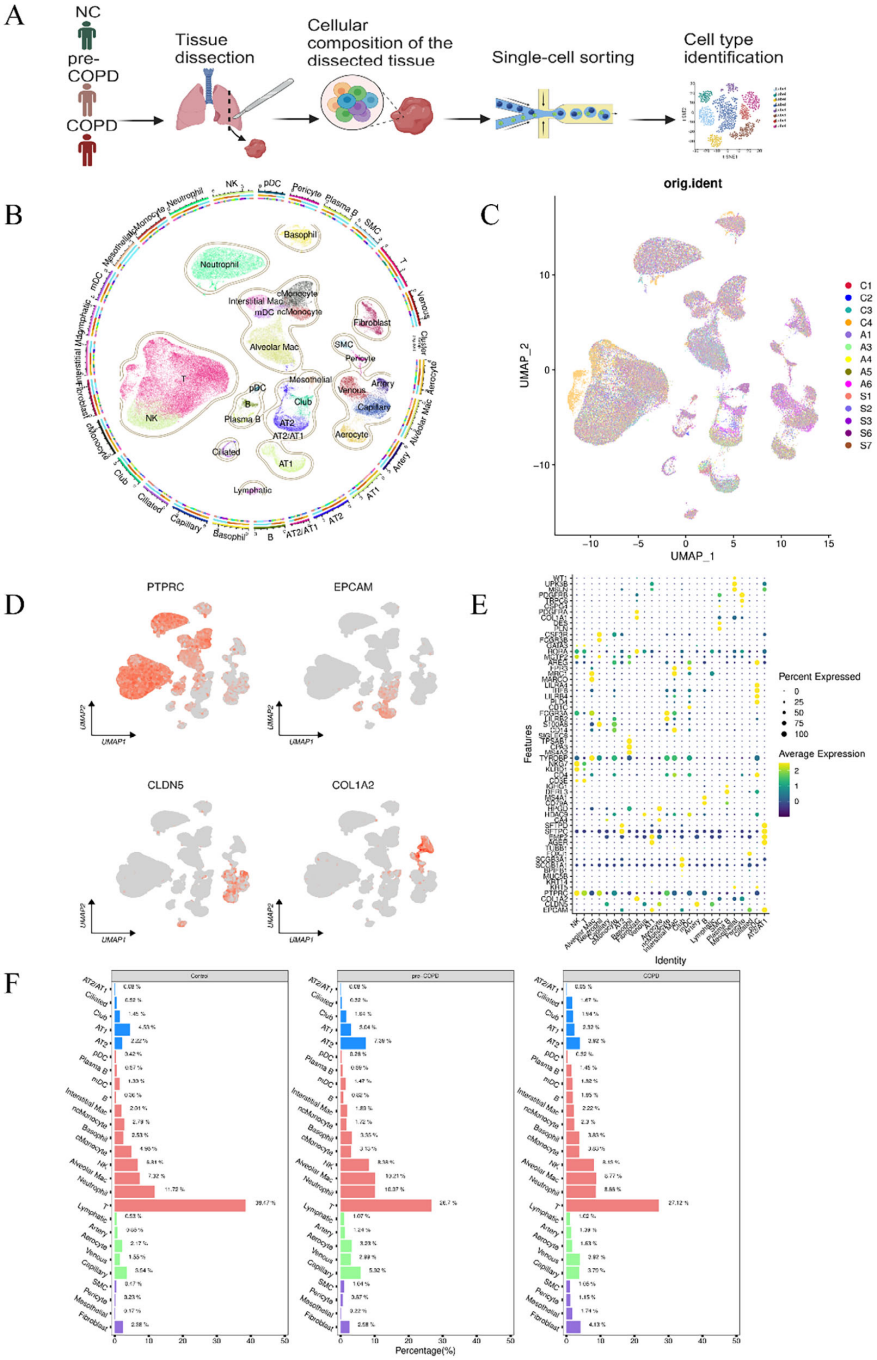
Single‐cell RNA sequencing reveals a comprehensive cell‐Type Atlas of COPD patients. Single‐cell RNA sequencing was performed on lung tissue isolated from COPD patients to construct a detailed cellular atlas. (A) Flowchart outlining the experimental design and analytical framework. (B) A total of 26 distinct cell types were annotated based on gene expression profiles. (C) UMAP visualization of cells from COPD and control donor lungs, colored by sample ID (Control: C1, C2, C3, C4; pre‐COPD: A1, A3, A4, A5, A6; COPD: S1, S2, S3, S6, S7). (D) Gene expression maps highlighting key marker genes for immune (*PTPRC* ^+^), epithelial (*EPCAM* ^+^), endothelial (*CLDN5* ^+^), and stromal (*COL1A2* ^+^) cell populations. (E) Dot plot illustrating the mean expression levels of marker genes clustered by cell type, as reported in the literature. (F) Quantification of the relative abundance of each cell subpopulation across different patient groups: Control (Ctl), pre‐COPD (A), and COPD.

We performed scRNA‐seq analysis on lung tissue samples from 4 control never‐smokers with normal lung function, 5 patients with pre‐COPD [pre‐COPD [20] was diagnosed in patients presenting with respiratory symptoms and small airways dysfunction (SAD) [21] but not diagnosed with COPD (i.e., postbronchodilator FEV1/FVC ≥ 0.70) or asthma], and 5 patients with established COPD (Figure 1A). Detailed clinical information for each patient is provided in Table S1 (Supplemental Methods), with pre‐COPD and stable COPD defined based on established criteria from the literature [22, 23]. To avoid the effects of tumor cells, which were present in the tissue samples from both pre‐COPD and stable COPD patients, we utilized the InferCNV tool to assess chromosomal aberrations including copy number variations (CNVs) which might suggest the presence of tumor cell contamination. No significant CNV events were observed in any sample (Figure S1), and no chromosomal copy number differences were detected among subpopulations within each group, ruling out the influence of tumor cells on the data.

Following quality control and removal of doublets, we retained a total of 40 834 cells from the control group, 47 802 cells from the pre‐COPD group, and 39 900 cells from the stable COPD group. Using Uniform Manifold Approximation and Projection (UMAP) for dimensionality reduction and clustering analysis, 26 distinct cellular clusters were identified (Figure 1B‐E). These clusters were subsequently annotated based on the expression of canonical marker genes [24].

To investigate potential differences in the relative numbers of immune cells in the samples from each group, we first calculated the proportions of each cell type within each group. T cells were the predominant immune cell type, with a noticeable decline in their relative abundance from the control group to the stable COPD group (mean 38.47%–27.12%) (Figure 1F).

### 2.2 Discovery and Dynamic Changes of MT‐High T Cells in COPD Progression

**FIGURE 2:**
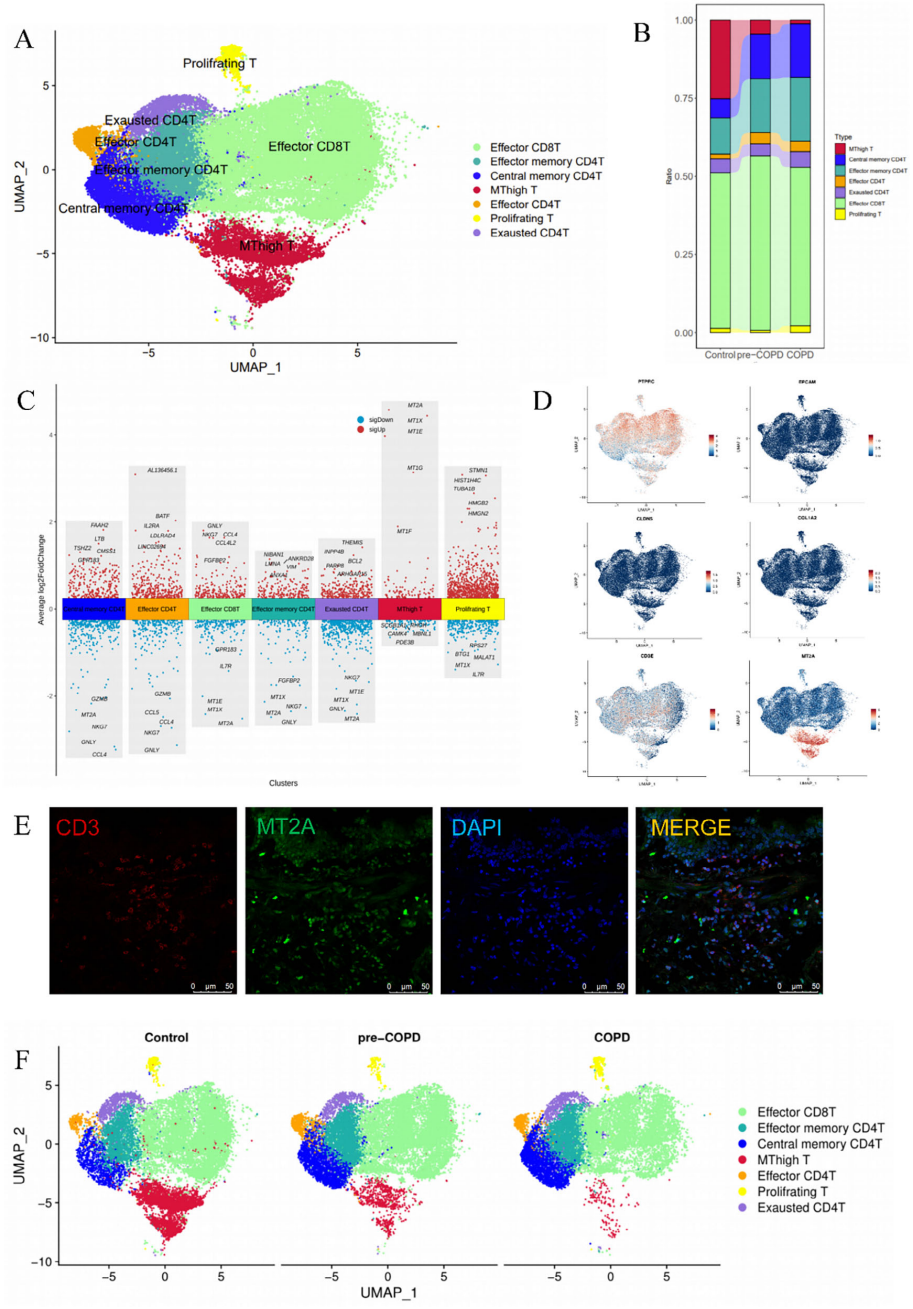
Characterization of T cell subpopulations in COPD using single‐cell RNA sequencing. (A) UMAP plots depicting T cells colored by their respective subclusters. (B) Sankey diagram showing the distribution of T cell subpopulations and their relative proportions across different groups. (C) Volcano plots highlighting the top five highly expressed (red) and least expressed (blue) genes in distinct T cell subpopulations. (D) Gene expression maps illustrating the expression of key markers for immune cells (PTPRC), epithelial cells (EPCAM), endothelial cells (CLDN5), stromal cells (COL1A3), T cells (CD3D), and metallothionein (MT2A). (E) Immunofluorescence staining of lung tissue sections from control subjects, with CD3D labeled in red, MT2A in green, and nuclei in blue. (F) UMAP visualization of MT‐high T cells in the control, pre‐COPD, and COPD groups.

We next focused on a detailed subpopulation analysis of T cells, the predominant immune cell type in the scRNA‐seq dataset. T cell subpopulations were annotated using gene expression distribution maps and dot plots based on established clustering markers [24] (Figure S2). Seven distinct T cell clusters were identified, including effector CD4^+^T, effector memory CD4^+^T, central memory CD4^+^T, exhausted CD4^+^T, effector CD8^+^T, and proliferating T (Figure 2A). Notably, we observed a trend toward increased proportions of effector CD4^+^T, effector memory CD4^+^T, central memory CD4^+^T, and effector CD8^+^T cells in pre‐COPD patients (Figure 2B). This finding contrasts with the well‐documented trend for enhanced T cellular infiltration in COPD [25], where such increases are typically more pronounced in advanced stages of the disease.

An intriguing observation in our analysis was the identification of a previously uncharacterized subpopulation of double‐negative T cells (DNT, i.e., CD3^+^CD4^−^CD8^−^). In order further to characterize these cells, we employed the Find Markers function and Volcano plots to identify significant upregulated genes in these cells, which revealed significant enrichment of metallothionein (MT)‐related genes, including MT1X, MT1E, MT1F, and MT2A (Figure 2C,D and Figure S3). Metallothioneins (MTs) are known regulators of intracellular zinc homeostasis, with MT1 typically expressed in fibroblasts, epithelial, and endothelial cells, where it acts as a heavy metal detoxifier in the cytoplasm. MT2A, by contrast, is predominantly expressed in the nucleus and cytoplasm and is implicated in cellular processes, such as proliferation and apoptosis. The existence of a subpopulation of T cells with high metallothionein expression has not, so far as we are aware, been described previously. Consequently, we labeled this subpopulation “MT‐high T cells.”

To validate the existence of MT‐high T cells, we performed immunofluorescence staining on paraffin sections of lung tissue from control subjects. Colocalization of CD3 (a T cell marker) and MT2A in lung tissue sections confirmed the presence of these cells in situ (Figure 2E). Additionally, we examined public scRNA‐seq datasets (Stable for data), which independently corroborated the existence of MT‐high T cells (Figure S4). Interestingly, we found that MT‐high T cells were significantly reduced in both pre‐COPD (4.52%) and stable COPD (1.16%) patients compared to control subjects (25.20%), with a progressive decline as the disease advanced (Figure 2F). This trend was consistent across all COPD patients, with MT‐high T cells either reduced or entirely absent in early and more advanced disease. In view of the fact that airway damage is thought to be effected in COPD at least partly by CD4^+^, Th1 effector, and cytolytic CD8^+^ T cells [26], we consider it likely that MT‐high T cells function as negative immune regulators in the lung microenvironment. The loss of these cells during disease progression may contribute to immune dysregulation in COPD, marking MT‐high T cells as a potential biomarker for disease status and progression.

### 2.3 MT‐High T Cell Deficiency as a Marker of Susceptibility to COPD

The above findings showing a progressive decline in the percentages of MT‐high T cells in the airways of patients with pre‐COPD progressing to COPD compared with control never‐smokers with normal lung function is consistent with the hypothesis that a deficiency of these cells predisposes to the development of pre‐COPD and COPD, with the corollary that such a deficiency may serve as a diagnostic indicator of disease susceptibility.

**FIGURE 3:**
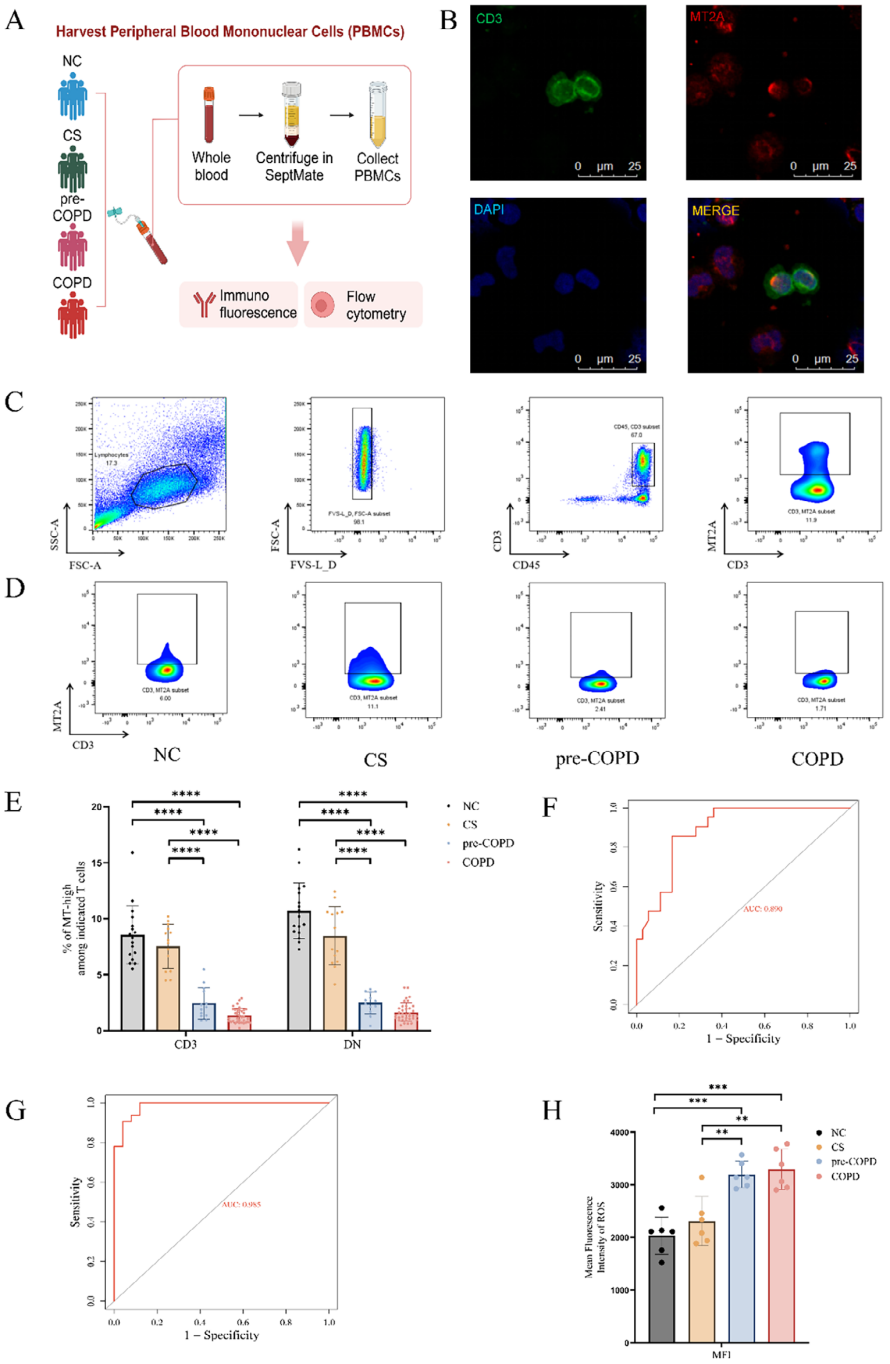
Validation of MT‐high T cells in the peripheral blood. (A) Experimental design and clinical sample collection for the validation of MT‐high T cells in peripheral blood. (B) Immunofluorescence images of peripheral blood mononuclear cells (PBMCs) from the control group, showing CD3D (green), MT2A (red), and nuclear staining (blue). (C) Flow cytometric analysis of PBMCs from healthy controls, depicting the gating strategy for lymphocytes, live cells, CD45^+^CD3^+^ T cells, and MT2A^high^ T cells. (D) Representative flow cytometry profiles of MT‐high T cells in peripheral blood from normal controls (NC), cigarette smokers (CS), pre‐COPD, and COPD patients. (E) Graph depicting the percentages of MT‐high T cells within CD3^+^ T cells and DNT cells across the NC, CS, pre‐COPD, and COPD groups. The mean percentages in the various groups were NC: CD3^+^: 8.56%, DNT: 10.69%; CS: CD3^+^: 7.54%, DNT: 8.48%; pre‐COPD: CD3^+^: 2.45%, DNT: 2.50%; COPD: CD3^+^: 1.36%, DNT: 1.65%. (F) ROC curve analysis demonstrates the high diagnostic and predictive accuracy of MT‐high T cell frequency for the standard COPD diagnostic criterion (FEV1/FVC < 0.7). (G) ROC curve analysis demonstrates the high diagnostic and predictive accuracy of MT‐high T cell frequency for pre‐COPD and COPD status. (H) Quantification of ROS mean fluorescence intensity in T cells across the NC, CS, pre‐COPD, and COPD groups (*N* = 6). Data represent mean ± SD. Statistical significance was determined by one‐way ANOVA with Tukey's post‐hoc test for multiple comparisons; **p < 0.01, ***p < 0.001, ****p < 0.0001.

In order to address this hypothesis, we first established that these cells can be identified in the peripheral blood. Peripheral blood mononuclear cells from three healthy subjects were collected and processed. We were able clearly to identify MT2A^+^ T cells in these samples using immunofluorescence, and a population of CD45^+^CD3^+^ T cells expressing high levels of MT2A using flow cytometry (Figure 3A–C and Figure S5).

We then measured the percentages of MT‐high T cells in the peripheral blood of further patient cohorts defined as described previously [20, 21, 23] (Supporting Information Methods): 17 healthy controls (NC, mean FEV1/FVC = 78.47%), 14 smokers with normal lung function (CS, FEV1/FVC = 76.00%), 13 patients with pre‐COPD (pre‐COPD, FEV1/FVC = 72.27%), and 36 patients with more advanced COPD (COPD, FEV1/FVC = 58.99%). Further details of these patients are provided in Table S2. Flow cytometry revealed a robust population of MT‐high T cells in the NC and CS groups. In contrast, this population was markedly reduced or absent in both the pre‐COPD and COPD groups (Figure 3D). Statistical analysis revealed that the mean percentages of CD3^+^MT‐high T cells and DNT‐MT‐high T cells were significantly lower in patients in both pre‐COPD (2.45%, 2.50%) and COPD (1.36%, 1.65%) groups compared with the NC (8.56%, 10.69%) and CS (7.54%, 8.48%) groups (Figure 3E). These findings are consistent with our scRNA‐seq data from lung tissue shown above, and further support our hypothesis that MT‐high T cells are a diagnostic biomarker for the early onset of COPD.

Furthermore, to rigorously evaluate the biomarker potential of MT‐high T cells (Figures S6 and S7), we conducted Receiver Operating Characteristic (ROC) curve analysis. The results were compelling: the MT‐high T cell frequency demonstrated excellent predictive power for both lung function decline (Figure 3F, AUC = 0.89) and the diagnosis of COPD (Figure 3G, AUC = 0.985). This provides strong statistical evidence that a low frequency of MT‐high T cells is a powerful indicator of disease presence and risk of progression.

The flow cytometric analysis of peripheral blood from COPD patients also revealed a significant depletion of MT‐high T cells in both pre‐COPD and more advanced stages of the disease. Previous studies have shown that MT2A is a potent antioxidant, capable of neutralizing reactive oxygen species (ROS) more effectively than glutathione (over 340 times more efficient). Based on this, we hypothesized that the decline in MT‐high T cells observed in COPD patients might be at least partly attributable to overwhelming of the antioxidant capacity of these cells owing to the accumulation of ROS, resulting in their apoptosis. To test this hypothesis, we assessed ROS levels in peripheral blood T cells from the same cohort. The analysis (Figure 3H) demonstrated a significant increase in the expression of ROS in T cells from both pre‐COPD and more advanced‐stage COPD patients compared to those from the NC and CS groups (*N* = 6), suggesting that the progressive accumulation of ROS during the progression of COPD does indeed result in depletion of MT‐high T cells by oxidative stress‐induced apoptosis.

### 2.4 MT‐High T is a Novel Subcluster of DNT Cells with Negative Regulatory Functions

**FIGURE 4:**
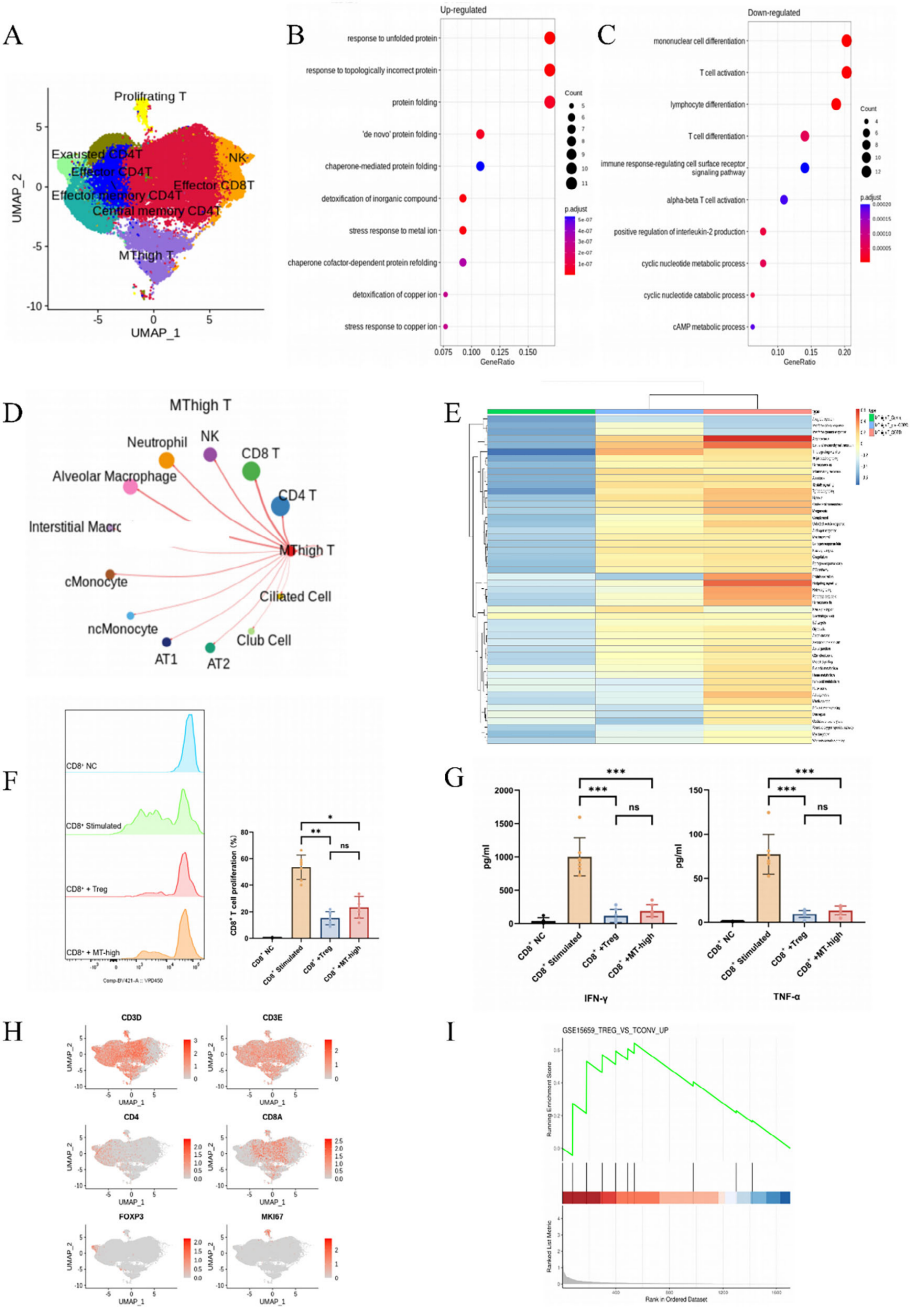
Functional profiling of gene expression s in MT‐high T cells. (A) UMAP visualization of T cell clusters. (B) Gene ontology (GO) analysis showing upregulated biological activities in MT‐high T cells. (C) GO analysis of downregulated biological activities in MT‐high T cells. (D) Chord diagrams illustrating the signaling interactions between MT‐high T cells and other immune and epithelial cells. Chords are color‐coded based on the origin (signal sender) or target (signal receiver) of the ligand–receptor pairs. (E) Gene Set Variation Analysis (GSVA) depicting the immunological functions of MT‐high T cells across different patient groups. (F) Proliferation of CD8^+^ T cells is inhibited by coculture with MT‐high T cells. VPD450‐labeled peripheral blood CD8^+^ T cells were stimulated with anti‐CD3/CD28 antibodies and cocultured with either Tregs (positive control) or MT‐high T cells. Representative histograms (left) and quantification (right) demonstrate that MT‐high T cells significantly suppress CD8^+^ T cell proliferation. Data represent the mean ± SD from seven independent donors; Data were analyzed using a one‐way repeated measures ANOVA followed by Tukey's multiple comparisons test. NS, not significant; * *p* < 0.05; ** *p* < 0.01. (G) ELISA quantification of IFN‐γ (left) and TNF‐α (right) concentrations in culture supernatants. Coculture with MT‐high T cells significantly inhibits the secretion of proinflammatory cytokines by activated CD8^+^ T cells. Data are presented as mean ± SD from seven independent donors. Statistical significance was determined by one‐way ANOVA with Tukey's post‐hoc test for multiple comparisons. NS, not significant; *** *p* < 0.001. (H) Feature plots of Treg cell‐associated marker gene expression (CD3D E: T cells; CD4: CD4^+^ T cells; CD8A: CD8^+^ T cells; FOXP3: Treg cells; MKI67: proliferation marker). (I) Gene set enrichment analysis (GSEA) was used to assess the correlation of DEGs in MT‐high T cells in relation to DEGs in Treg cells. The green line represents the Enrichment Score (ES). The black lines represent genes in the examined gene set. The red region signifies positive correlation, whereas the blue region signifies negative correlation.

Having identified the potential of MT‐high T cells to serve as an early marker for the potential development of COPD, we next sought to explore their functional role in disease progression. We performed functional enrichment analyses of the differentially expressed genes (DEGs) in MT‐high T cells (Figure 4A). Gene ontology (GO) enrichment analysis revealed that MT‐high T cells exhibited elevated expression of genes involved in protein folding, detoxification of inorganic compounds and the stress response to metal ions, which is consistent with the physiological role of MTs. Additionally, the T cell activation pathway was also enriched, suggesting that MT‐high T cells are in an activated state (Figure 4B). Conversely, the DEGs with lower expression were predominantly associated with the down‐regulation of pathways related to positive cellular regulation, such as T‐cell differentiation, positive T‐cell activation, and positive regulation of cytokine production (Figure 4C). This pattern of gene expression supports the hypothesis that MT‐high T cells function as negative immune regulators, possibly dampening excessive immune activation.

To further elucidate the immunomodulatory role of MT‐high T cells, we analyzed their cell–cell interactions with other immune and epithelial cells within the disease microenvironment. Using the CellChat tool, we identified strong potential interactions between MT‐high T cells and neutrophils, alveolar macrophages and other T cells (Figure 4D), again consistent with the hypothesis that MT‐high T cells play a significant role in modulating immune responses so as to inhibit COPD progression. To explore the temporal dynamics of MT‐high T cell function, we conducted Gene Set Variation Analysis (GSVA) on differentially expressed genes (DEGs) from MT‐high T cells across the stages of COPD progression. The results (Figure 4E) revealed significant enrichment in several pathways, including inflammatory response, apoptosis, hypoxia, and TGF‐β in MT‐high T cells in patients with pre‐COPD and more advanced COPD. These findings indicate that MT‐high T cells are involved in both inflammatory and oxidative stress responses, further supporting their role in COPD pathogenesis. In addition, comparative analysis across patient groups revealed striking functional alterations in CD8^+^ T cells that correlated with MT‐high T cell abundance. Specifically, CD8^+^ T cells from pre‐COPD and COPD patients exhibited a significant upregulation of cytokine production pathways, particularly TNF‐α signaling, compared with those from controls (Figure S10). Building upon these computational insights, we proceeded with direct functional validation through ex vivo coculture experiments. MT‐high T cells were isolated from the peripheral blood of seven healthy volunteers via flow cytometric sorting and cocultured with CD8^+^ T cells activated by anti‐CD3/CD28 stimulation. The results demonstrated that MT‐high T cells possess potent suppressive activity (Figure 4F). In the presence of MT‐high T cells, the secretion of key proinflammatory cytokines (IFN‐γ and TNF‐α) by activated CD8^+^ T cells was significantly inhibited (Figure 4G). These data confirm that MT‐high T cells are not merely a marker of disease susceptibility but are functionally capable of restraining CD8^+^ T cell effector responses.

Given the potential immunomodulatory functions of MT‐high T cells, we examined whether they might represent a subtype of regulatory T cell (Treg). Expression of the Treg marker gene FOXP3 was assessed, and the results demonstrated that MT‐high T cells were predominantly CD3^+^CD4^−^CD8^−^FOXP3^−^, a phenotype characteristic of DNT cells, rather than conventional Tregs (Figure 4H and S3). To further investigate this, we performed Gene Set Enrichment Analysis (GSEA) comparing the gene expression profiles of MT‐high T cells and Tregs (Figure 4I). Only seven genes were upregulated in both populations, indicating that MT‐high T cells do not operate through the conventional immunomodulatory mechanisms of Tregs. Instead, these cells likely represent a novel subtype of double negative (CD4^−^CD8^−^) T cells with distinct immunomodulatory functions.

### 2.5 MT‐High T Cells Regulate CD8^+^ T Cell Proliferation, Activation, and Cytotoxicity Through MIF Signaling Pathways

**FIGURE 5:**
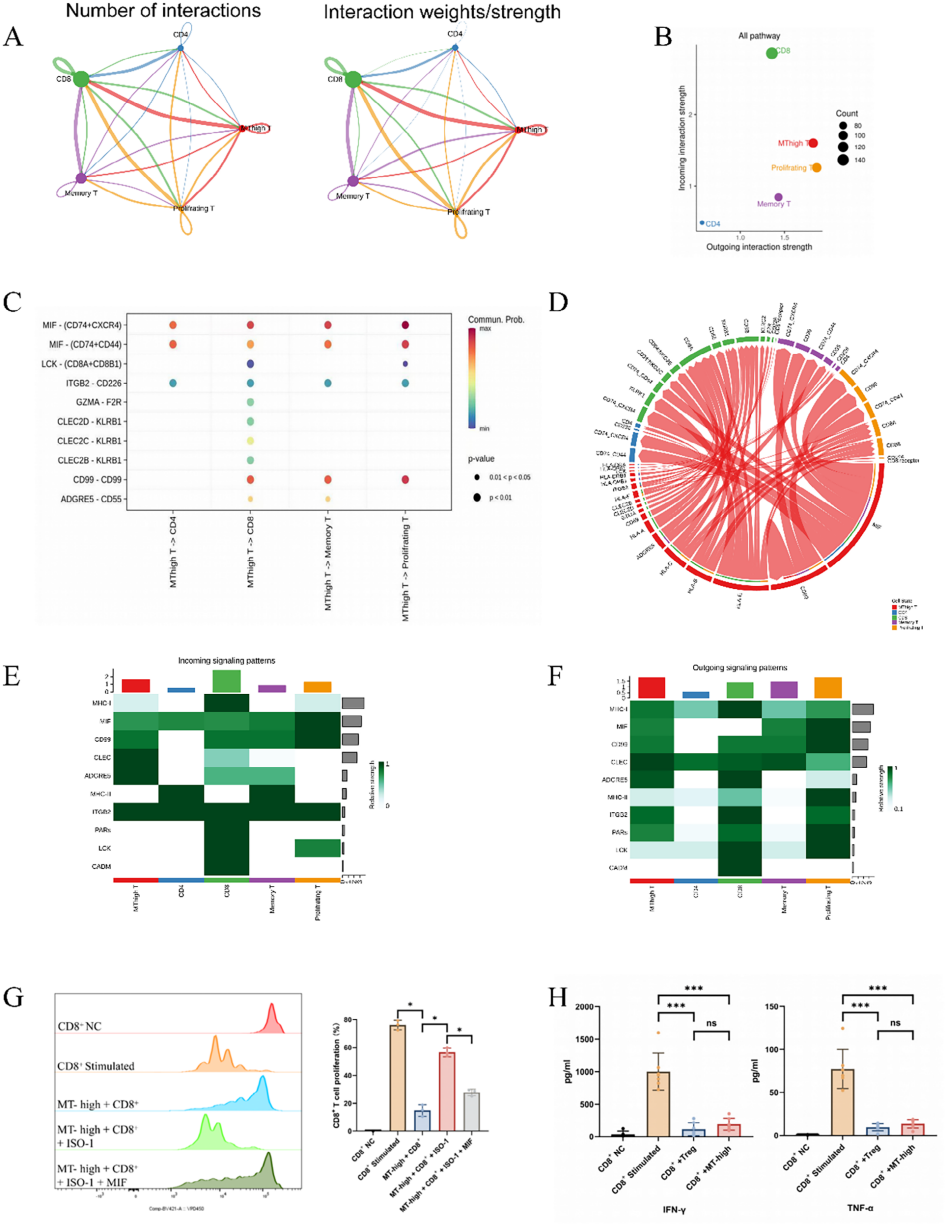
T cell networks and pathway centrality analyses. (A) Number of ligand–receptor pairs and strength of cellular communication between T cell clusters. This panel shows the ligand–receptor interactions detected between different T cell subpopulations. Solid circles represent distinct cell groups, with the size of the circle proportional to the number of cells in that group. The color of each edge corresponds to the signal sender, and the width of each edge reflects the strength of the communication. (B) Inference of T cell subset roles. Each dot represents a T cell subset, with the color indicating the specific cell group. The size of the dot is proportional to the number of ligands and receptors inferred for that subset. The *x*‐axis and *y*‐axis represent the strength of the subset as a signal sender and receiver, respectively. (C) Dot plots showing ligand–receptor interactions that mediate communication between MT‐high T cells and other T cell clusters. (D) Prediction of signaling pathways involved in coordinated responses among T cell subpopulations. The cell groups and signaling pathways are represented by nodes, and the thickness of the flow indicates the contribution of each cell group or signaling pathway to the communication pattern. This illustrates how cells coordinate as signal senders in outgoing communication patterns and interact with specific signaling pathways to facilitate these communications. (E,F) Heatmap of the signaling network between T cell subpopulations. These panels show the signaling pathways received (incoming) or emitted (outgoing) by each T cell subset. The color intensity represents the strength of the signal. (G) Functional validation of the MIF pathway in proliferation suppression. Representative histograms (left) and quantification (right) of CD8^+^ T cell proliferation. The suppressive effect of MT‐high T cells is reversed by the MIF inhibitor ISO‐1 and is restored by the addition of rhMIF. Data are presented as mean ± SD from three independent donors. Statistical significance was determined by one‐way repeated measures ANOVA with Tukey's post hoc test for multiple comparisons. * *p* < 0.05. (H) Functional validation of cytokine suppression. ELISA quantification of IFN‐γ (left) and TNF‐α (right) concentrations. MT‐high T cells significantly inhibit the secretion of proinflammatory cytokines from activated CD8^+^ T cells in an MIF‐dependent manner. Data are presented as mean ± SD from three independent donors. Statistical significance was determined by one‐way repeated measures ANOVA with Tukey's post hoc test for multiple comparisons. NS, not significant; *** *p* < 0.001.

Functional analysis of MT‐high T cells revealed their potential role as immunoregulatory cells, with the most intense interactions observed between MT‐high T cells and various T cell subsets (Figure 4D). To further explore these interactions, we constructed an intercellular communication network using the CellChat tool (Figure 5A). Our analysis revealed that the interaction between MT‐high T cells and CD8^+^ T cells was the most pronounced, in terms of both the numbers and the intensities of the ligand–receptor interactions. This suggests that MT‐high T cells exert a dominant role in modulating CD8^+^ T cell activity. In‐depth network analysis, further confirmed that MT‐high T cells predominantly serve as signal senders, while CD8^+^ T cells are the major receivers of these signals (Figure 5B). Immunofluorescence staining of lung tissue (Figure S8) indicated the spatial proximity of CD8^+^ T cells and MT‐high T cells. This phenomenon suggested the potential for MT‐high T cells to regulate CD8^+^ T cells and validated the findings from our scRNA‐seq data.

Focusing on the specific ligand–receptor pairs involved in these interactions, we identified MIF‐(CD74+CXCR4) as the most potent signaling axis between MT‐high T cells and CD8^+^ T cells (Figure 5C). Examination of ligand–receptor chordograms (Figure 5D) highlights that MT‐high T cells are the major contributors to the MIF signaling pathway, which predominantly targets CD8^+^ T cells. Additionally, heatmaps (Figure 5E, F) revealed that CD8^+^ T cells are prominent receivers of MIF signals, further emphasizing their key role in this immune interaction. To verify this, we first confirmed that MT‐high T cells are an important source of Macrophage migration inhibitory factor (MIF). Using ELISA, we measured MIF protein in the supernatants of different cell cultures. As shown in Figure S9, MIF concentrations were significantly elevated in cultures containing MT‐high T cells (both alone and in coculture with CD8^+^ T cells) compared with cultures of CD8^+^ T cells alone. In contrast, MT‐high T cells were identified as the primary emitters of MIF signaling.

Previous studies have shown that MIF can suppress the activation and proliferation of autoreactive CD8^+^ T cells [27]. In the current study, we designed a series of experiments to prove that the suppressive effect of MT‐high T cells is dependent on MIF. The activated T cells were then cocultured with MT‐high T cells in the presence or absence of a specific MIF inhibitor (ISO‐1) and recombinant human MIF (rhMIF). The inhibitory effect of MT‐high T cells on proliferation and cytokine production was significantly reversed by the addition of the MIF inhibitor ISO‐1. Furthermore, adding back recombinant MIF to the ISO‐1‐treated cocultures restored this suppressive effect (Figure 5G, H). This observation is consistent with the hypothesis that MT‐high T cells inhibit CD8^+^ T cell activation and proliferation through MIF secretion, thereby exerting a negative regulatory effect on CD8^+^ T cell responses.

Taken together, these data suggest that MT‐high T cells regulate CD8^+^ T cell proliferation, activation and cytotoxicity through multiple signaling pathways, including MIF. The progressive decline in the proportion of MT‐high T cells observed in COPD may lead to unchecked CD8^+^ T cell expansion and sustained inflammatory responses in the early stages of the disease. This imbalance could contribute to the chronic, irreversible inflammation that characterizes COPD progression.

### 2.6 Differentiation and Fate of MT‐High T Cells in COPD

**FIGURE 6:**
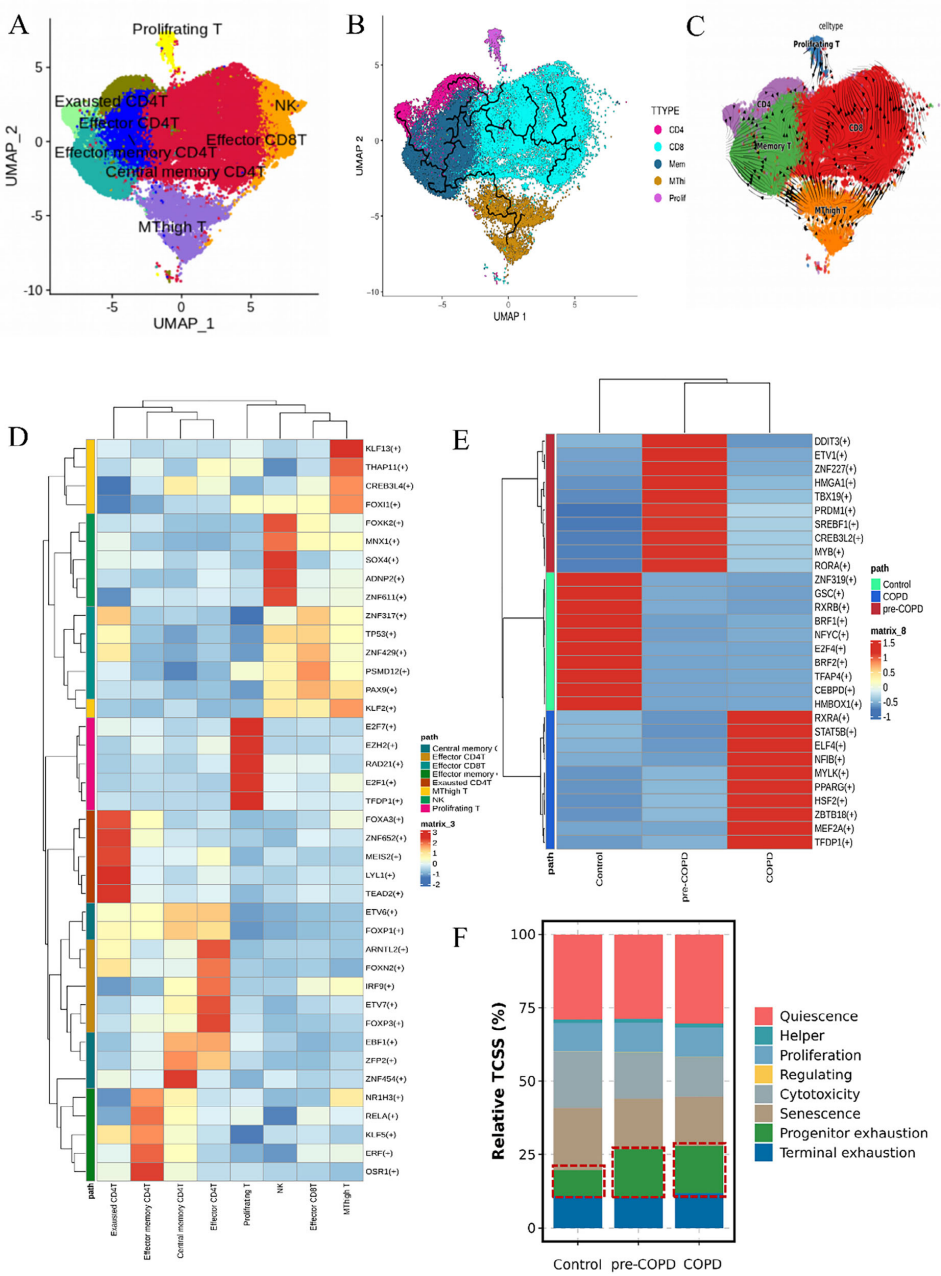
MT‐high T cell trajectory analysis and prediction of critical transcription factors in gene regulatory networks. (A) UMAP visualization of T cells. (B) Trajectory analysis of T cells using Monocle3. Memory T cells, which are dark blue, have been identified as the starting point of the developmental trajectory in our study, consistent with established models of T cell development. (C) Trajectory analysis of T cells based on RNA velocity. The direction of the arrows indicates the predicted developmental trajectory of T cells, with arrow density reflecting the instantaneous rate of transcriptional changes. (D) Heat‐map of the *t* values of area under the curve (AUC) scores of expression regulation by transcription factors of the MT‐high T clusters, as estimated using pySCENIC. (E) Heat‐map of the *t* values of AUC scores of expression regulation by transcription factors of the MT‐high T clusters in different groups, as estimated by pySCENIC. (F) Assessment of functional state changes of MT‐high T cells between groups using T Cell State Identifier (TCell SI).

To investigate the origin and fate of MT‐high T cells, we utilized the Monocle3 and RNA velocity tools to infer the proposed temporal developmental trajectories of distinct clusters of T cells. From a priori knowledge, the starting point of the proposed temporal trajectory was identified as memory T cell, located in the lower left corner of the UMAP plot (Figure 6A, B). From this initial state, the analysis revealed three distinct T cell differentiation pathways: memory T→MT‐high T; memory T→effector CD4^+^ T; and memory T→effector CD8^+^ T. Using RNA velocity, we observed that memory T cells exhibited a directional arrow pointing toward MT‐high T cells (Figure 6C). This suggests that memory T cells have a propensity to differentiate into MT‐high T cells. The consistency across multiple trajectory analysis methods supports the hypothesis that MT‐high T cells arise from memory T cells in the lung, representing a distinct branch of differentiation from both CD4^+^ and CD8^+^ T cells. Further validation of this model was obtained by leveraging developmental scRNA‐seq data of healthy thymic T cells from Park et al. [28], which revealed specific expression of MT2A in double‐negative and some single‐positive T cells (Figure S15). This finding corroborates our observations on MT‐high T cells in lung tissue and suggests a potential original thymic origin for these cells, which could subsequently differentiate from tissue‐resident memory T cells in the lung.

To explore the factors driving the emergence of MT‐high T cells and their reduction in disease states, we analyzed the transcription factor profiles specific to the MT‐high T cell cluster and disease phases using the pySENIC tool. We identified a transcription factor—KLF13—that was significantly activated in the MT‐high T cell subset compared to other T cell subsets (Figure 6D). Notably, KLF13 was strongly implicated in immune cell differentiation and development, suggesting that this transcription factor plays a pivotal role in determining the fate of MT‐high T cells. In the control group, MT‐high T cells exhibited upregulation of several transcriptional activators, including ZNF319, RXRB, and BRF1 (Figure 6E). The significant activation of these transcriptional activators may explain the higher abundance of MT‐high T cells in our control groups of patients compared to those with pre‐COPD and more advanced COPD.

To further explore the functional status of MT‐high T cells in COPD, we applied the T Cell State Identifier (TCell SI) tool, developed by Guo and colleagues [29], to assess various T cell states. Our analysis (Figure 6F) suggested an association between the development and progression of COPD with a progressive depletion of MT‐high T cells. Depletion of MT‐high T cells may disrupt the delicate balance of CD8^+^ T cell regulation, contributing to enhanced tissue damage through the cytotoxic effects of CD8^+^ T cells, which may in turn contribute to the irreversible damage to lung parenchyma and small airways characteristic of progressive COPD.

## 3 Discussion

COPD is a highly complex and heterogeneous condition, characterized by a wide range of clinical phenotypes and varying disease trajectories. One of the most critical deficiencies in current clinical management is the lack of criteria to recognize patients at risk of developing the disease and intervene in its progression before symptoms appear. To address this challenge, we employed single‐cell RNA sequencing to create a comprehensive cellular atlas of lung tissue from patients with normal lung function, pre‐COPD, and stable COPD. This analysis revealed a novel T cell subset, which we termed MT‐high T cells.

This previously unidentified subpopulation of T cells is characterized by markedly elevated expression of metallothionein genes, which are known to protect against oxidative stress. Importantly, the abundance of these cells showed a progressive decline: they were most elevated in control subjects, reduced in patients with pre‐COPD, and further diminished in those with established disease. This pattern strongly suggests these cells are functionally involved in preventing disease development and progression.

Metallothioneins (MTs) are a family of cysteine‐rich metal‐binding proteins primarily involved in regulating metal ion homeostasis and mitigating oxidative stress [30]. Established literature confirms that MTs are expressed across diverse lung cell types, including epithelial and endothelial cells, where they provide broad, inducible cytoprotection against environmental insults, such as particulate matter [31, 32, 33]. Beyond this general protective role, our work reveals that the constitutive high‐level expression of a metallothionein gene program defines a functionally specialized T cell subtype. Critically, the disease‐relevant observation is that this uniquely equipped T cell population is specifically depleted during COPD progression, suggesting a loss of protective immune capacity that may contribute to disease pathogenesis.

Our functional enrichment analysis revealed that MT‐high T cells exhibit negative immunomodulatory functions, potentially contributing to the regulation of immune responses in the lung microenvironment. These cells, characterized by the phenotype CD3^+^ CD4^−^ CD8^−^, appear to play a significant immunoregulatory role through their ability to inhibit the activation, proliferation, and cytotoxic activity of CD8^+^ T cells, partly through secretion of signaling molecules, such as MIF.

To validate our tissue‐based findings, we identified MT‐high T cells in peripheral blood samples from a clinical cohort of 80 individuals. Consistent with our lung tissue observations, we found a progressive reduction in the numbers of circulating MT‐high T cells in patients with pre‐COPD progressing to established COPD. This finding underscores their potential role as an early and readily quantifiable cellular marker of COPD susceptibility, enabling identification of at‐risk individuals before the onset of clinically overt symptoms.

Our data support the hypothesis that MT‐high T cells contribute to a protective mechanism that prevents or limits inflammatory changes relevant to COPD pathogenesis. When these regulatory cells become depleted, the resulting disruption of immunological balance leads to unchecked CD8^+^ T cell activity, heightened immune responses and subsequent tissue damage in the small airways. This scenario provides a novel and plausible explanation for the persistent infiltration of CD8^+^ T cells and chronic airway damage observed in advanced COPD.

Additionally, our cell trajectory inference and transcription factor prediction analysis suggests that MT‐high T cells likely differentiate from memory T cells (Tm), a subset of long‐lived immune cells central to immune memory and homeostasis. This finding will complement future studies investigating the origins and functional characteristics of MT‐high T cells in chronic diseases.

While these findings provide novel and critical insights into immune regulation, COPD pathogenesis and early disease identification, several areas warrant further investigation. Given the broad range of functions attributed to MIF [34, 35], detailed mechanistic studies are essential to elucidate how MT‐high T cells modulate MIF signaling pathways in COPD pathogenesis. Specifically, future research should focus on characterizing the precise molecular mechanisms through which these cells attenuate the chronic inflammation and tissue remodeling which ultimately result in irreversible airways obstruction. To definitively confirm the in vivo function of these cells, depletion and adoptive transfer studies would be ideal. A major challenge to this goal, however, is the absence of an appropriate animal model harboring a T cell subset analogous to human MT‐high T cells. To bridge this gap, we propose two strategic approaches for future investigation. First, we aim to develop murine surrogates engineered to express key human metallothionein family genes within the context of established COPD protocols (e.g., cigarette smoke exposure). This system will allow for the dissection of cell‐intrinsic functions in a disease‐relevant physiological setting. Second, we will pursue the adoptive transfer of patient‐derived, ex vivo expanded MT‐high T cells into immunodeficient mice with experimental COPD. We anticipate that these approaches will represent a crucial next step toward causally linking these cells to the modulation of immunopathology. Furthermore, our hypothesis that MT‐high T cell deficiency in both the peripheral circulation and the lung parenchyma predisposes individuals to COPD development requires validation through comprehensive translational studies. These should include lung‐specific functional assays, expanded cross‐sectional cohort studies with diverse populations and prospective longitudinal investigations to establish causality and determine the predictive value of MT‐high T cell enumeration in clinical practice. Such studies will be crucial for establishing the clinical utility of this biomarker and its potential integration into routine risk assessment protocols.

In conclusion, this study identifies enumeration of MT‐high T cells as a potential method for identifying individuals at risk of COPD prior to disease establishment, while uncovering novel immunological mechanisms of COPD pathogenesis for future research exploration. Our findings provide a comprehensive immune cell atlas of lung tissue in pre‐COPD patients, establishing a crucial theoretical and experimental foundation for better understanding the immunological mechanisms underlying pre‐COPD. These discoveries open new potential avenues for early identification of at‐risk patients and development of novel interventional strategies, representing a significant step toward personalized, preventive approaches to COPD management.

## 4 Experimental Section

### 4.1 Selection of Clinical Subjects, Lung Tissue Collection, and PBMC Isolation

Adult subjects aged between 35 and 80 years were recruited from four centers: The China‐Japan Friendship Hospital, The Eighth Medical Center of the Chinese PLA General Hospital, Peking University Third Hospital and the Beijing Xuanwu Hospital. Lung tissue samples were obtained from three groups of patients: four control never‐smokers with normal lung function, five subjects with pre‐COPD, and five subjects with established COPD. The patients were recruited according to established criteria [22, 23]: Specifically, the inclusion criteria were as follows: (1) Healthy Controls: Participants had no history of asthma, COPD, or any acute or chronic infectious respiratory diseases. Pulmonary function tests (pre‐ and postbronchodilator) showed forced expiratory volume in 1 s (FEV1) > 80% of the predicted value, and the FEV1/forced vital capacity (FVC) ratio > 0.8. (2) pre‐COPD: People who presented with respiratory symptoms (including chronic cough, chronic sputum production, dyspnea, and/or exacerbation) and SAD but not diagnosed with COPD (i.e., postbronchodilator FEV1/FVC ≥ 0.70) or asthma. The criteria used to define SAD were consistent with a prior analysis in the CPH study [21]. (3) Stable‐Stage COPD: Participants were diagnosed with COPD based on pulmonary function tests showing an FEV1/FVC ratio < 0.7 and an FEV1< 80% of the predicted value.

Biological samples were collected from the lung tissues and peripheral blood of the participants. Lung tissue specimens from the healthy control, pre‐COPD, and stable‐stage COPD groups were all collected from patients undergoing lung cancer surgery, ensuring that the specimens were taken from areas at least 3 cm away from the tumor site and pathologically free of cancerous cells. These lung tissue samples were utilized for single‐cell sequencing analysis.

Blood samples for peripheral blood mononuclear cell (PBMC) isolation were obtained from eligible participants at the specified time points. The cohort included four groups: 17 nonsmokers, 14 healthy heavy smokers, 13 subjects with pre‐COPD, and 36 subjects with stable‐stage COPD. All participants provided written informed consent in accordance with the ethical guidelines approved by the Ethics Committee of Capital Medical University (Ethics Approval Number: Z2021SY025). Subjects were all aged between 35 and 80 years. The inclusion criteria for each group complied with internationally‐recognized guidelines [22, 23]: (1) Nonsmoking control group (NC): Controls were defined as people who were not smoking, with pre‐ and postbronchodilator FEV_1_/FVC ≥ 0.70, FEV1 ≥ 80% of the predicted value, and not diagnosed with asthma. (2) Smoking control group (CS): Controls were defined as smokers (> 50 pack years), with pre‐ and postbronchodilator FEV_1_/FVC ≥ 0.70, FEV1 ≥ 80% of the predicted value, and not diagnosed with asthma. (3) Pre‐COPD group: Pre‐COPD patients were defined as those presenting with respiratory symptoms (including chronic cough, chronic sputum production, dyspnea, and/or exacerbation) and small airway dysfunction (SAD) but not diagnosed with COPD (i.e., postbronchodilator FEV1/FVC ≥ 0.70) or asthma. The SAD judging criteria were consistent with a prior analysis of the CPH study [21]. (4) Stable‐Stage COPD: Participants with pulmonary function tests showing FEV1/FVC < 0.7 and FEV1< 80% of the predicted value, consistent with stable COPD. Blood was drawn into EDTA and heparin‐coated serum vacuum tubes (BD Biosciences, Catalog Nos. 366643 and 668660) for subsequent processing. Plasma and serum were separated within 3 h of collection and stored appropriately for further analysis. PBMCs were isolated from the EDTA‐containing blood samples using Ficoll–Paque density gradient centrifugation.

### 4.2 scRNA‐seq Analysis of Human Lung Tissue Samples

scRNA‐seq was performed on human lung tissue samples to profile cellular diversity and gene expression. Live cells were first isolated using a fluorescence‐activated cell sorting (FACS) strategy, followed by preparation of single‐cell cDNA libraries using the Chromium Single Cell 3’Reagent v3 Kit (10X Genomics, Catalog No. 120234) according to the manufacturer's protocol. Cells were resuspended at a concentration of approximately 1 × 10^6^ cells/mL in PBS containing 0.1% BSA. Next, single‐cell suspensions were loaded into the Chromium instrument to generate single‐cell gel bead‐in‐emulsions (GEMs), where full‐length cDNA synthesis was performed. The resulting cDNA was purified, amplified, and fragmented. Subsequently, the libraries were tagged with a 3’‐junction and sample‐specific indexes to ensure proper barcoding. Library sequencing was conducted on the Illumina platform using 150‐bp paired‐end reads. The raw data were processed and demultiplexed into FASTQ files using the Cell Ranger mkfastq pipeline (v3.0.2). To ensure data quality, Read2 files were trimmed using Cutadapt (v2.7) to remove any reads shorter than 20 bp. For downstream analysis, the processed reads were aligned to the human reference genome (GRCh38.p12, version 91) using STAR (v2.6.0c). Cells were excluded from subsequent analysis if they met any of the following criteria: (1) fewer than 1000 detected transcripts, (2) greater than 20% mitochondrial‐derived transcripts, or (3) fewer than 12% of total transcript counts derived from high‐confidence, pruned mRNAs. Gene expression values were normalized to 10 000 transcripts per cell and log‐transformed using a dummy count of 1 to adjust for library size and sequencing depth, facilitating accurate comparison of transcriptomic profiles across samples.

### 4.3 scRNA‐seq Data Processing and Cell Type Annotation

The raw data from the Cell Ranger output were initially processed to remove doublets using DoubletFinder [36], with a doublet rate adjustment of 8 parts per thousand for every additional 1000 cells. Following doublet removal, data integration was performed using Seurat v4.0 [37]. To ensure data quality, cells with more than 10% mitochondrial gene content or extreme values for unique gene counts (fewer than 800 or more than 5000 genes per cell) were excluded from subsequent analysis. The remaining data were normalized using sctransform v0.3.2 [38] following quality control (QC) checks. To address potential batch effects, the data were integrated using Harmony v1.0 [39], which facilitated batch removal across different experimental conditions. Principal component analysis (PCA) was performed on the normalized data, and the first principal components were subsequently used for dimensionality reduction with uniform manifold approximation and projection (UMAP). Cell clusters were identified at a resolution of 0.5, which was selected based on the optimal separation of cellular populations. Cluster annotation was then performed using prior knowledge of cell‐type markers and published references to assign each cluster to its corresponding major cell type.

### 4.4 scRNA‐seq‐Based CNV Detection

Copy number variations (CNVs) in malignant cells were detected using the InferCNV software package [40]. To estimate CNVs, nonmalignant cells were used as a reference baseline. Genes expressed in at least 20 cells were considered for analysis and classified according to their chromosomal loci. Relative expression values were normalized and centered around 1, with the upper threshold set at 1.5 standard deviations (SD) from the residual normalized expression values. To mitigate the effects of gene‐specific expression variability, the relative expression across each chromosome was smoothed using a sliding window approach, with a window size of 101 genes. This approach ensured a more robust detection of CNVs by reducing noise from local gene expression fluctuations.

### 4.5 Pathway Enrichment Analysis

To investigate the functional implications of differentially expressed genes (DEGs), we performed gene ontology (GO) analysis using the “clusterProfiler” R package (version 4.10.0) [41]. Significantly enriched pathways were identified based on corrected *p*‐values (p_adj) below 0.05. The analysis included GO categories related to molecular function, biological processes, and cellular components. Additionally, gene set enrichment analysis (GSEA) was employed to assess the enrichment of DEGs between the two experimental groups. For further functional characterization, gene set variation analysis (GSVA) was applied to evaluate pathway enrichment at the cell type level. The average gene expression of each cell type was used as input for GSVA, allowing us to identify pathway activation profiles specific to distinct cellular populations.

### 4.6 Cell‐to‐Cell Interaction Analysis

Intercellular communication networks were quantitatively inferred and analyzed using the CellChat R package (version 1.6) [42]. This tool enables the identification of overexpressed ligands and receptors within specific cell populations and maps ligand–receptor interactions that are significantly enriched in the dataset. CellChat integrates prior knowledge of gene expression, signaling ligands, receptors, and their cofactors to model the probability of intercellular communication. By doing so, it provides a comprehensive view of the signaling networks between cells. To account for potential gaps in signaling data, particularly the absence of ligand or receptor subunits due to zero expression, CellChat overlays gene expression data onto a protein–protein interaction (PPI) network. This approach helps mitigate the impact of missing information, ensuring a more complete analysis of intercellular communication. To visualize the results, we employed hierarchical plots, circle plots, and bubble plots, which allowed us to highlight both autocrine and paracrine signaling interactions between the cell groups of interest. These visualizations provided an intuitive and detailed representation of the communication pathways active in our dataset.

### 4.7 Transcription Factor Regulator Activity

Transcription factor (TF) regulator activity was evaluated using the SCENIC algorithm (version 1.0.1) [43]. Briefly, SCENIC identifies key TF regulators by assessing the coexpression of TFs with their target genes, followed by motif enrichment analysis. The activity of each TF regulator was then quantified by calculating the AUCell score, which ranges from 0 to 1 and reflects the level of activity for each TF in individual cells. This score provides a robust measure of TF regulation, enabling the identification of active regulatory programs within the cellular landscape. To ensure accurate interpretation, regulator activity was assessed independently for different major cell types, allowing for cell‐type‐specific insights into TF regulation.

### 4.8 RNA Velocity and Pseudotime Analysis

RNA velocity analysis was performed using scvelo (version 0.1.25) in Python [44]. Following gene selection and normalization, we computed first‐ and second‐order moments using the scv.pp.moments function. In dynamics mode, we recovered full splicing dynamics with the scv.tl.recover_dynamics function. RNA velocity was subsequently calculated using the scv.tl.velocity function, providing a directional flow of transcriptional activity within individual cells. To visualize these dynamics, the velocity was projected onto a diffusion map and represented as a streamline using the scv.pl.velocity_embedding_stream function. Additionally, spliced and unspliced phase maps for individual genes were visualized with scv.pl.velocity. To explore cellular progression over developmental or differentiation trajectories, pseudotime was inferred using the scv.tl.recover_latent_time function, which estimates the pseudotime ordering of cells based on their RNA velocity. For further exploration of pseudotemporal trajectories, we also utilized Monocle3 [45], which provides a robust framework for analysis of changes in gene expression over time. Monocle3's differential expression analysis allows the identification of genes that exhibit dynamic expression patterns across cell types or developmental stages, thereby facilitating the construction of pseudotemporal trajectories.

### 4.9 Immunofluorescence Staining

Immunofluorescence staining was performed on 4 µm tissue sections of formalin‐fixed paraffin‐embedded (FFPE) tissues. The sections were deparaffinized, rehydrated, and subjected to antigen retrieval using sodium citrate buffer. Endogenous peroxidase activity was blocked, and sections were subsequently incubated with 10% normal goat serum for 30 min to minimize nonspecific binding. Primary antibody staining was conducted overnight at 4°C in a humidified chamber, using anti‐CD3 (Abcam, AB135372; 1:250) and anti‐MT2A (Affinity, DF6755; 1:100) antibodies. Following primary antibody incubation, sections were incubated with secondary antibodies: Goat anti‐Rabbit IgG (AF488, ZSGB‐BIO, ZF‐0511; 1:1000) and Goat anti‐Mouse IgG (AF594, ZSGB‐BIO, ZF‐0513; 1:1000) for 1 h at room temperature. Nuclei were counterstained with DAPI. Finally, stained slides were imaged and scanned using a Leica DM6000 CS orthogonal fluorescence microscope and analyzed by Leica LASX analysis software.

### 4.10 MT‐High T‐Cell Flow Cytometry Staining

Flow cytometry was performed using the BD Fortessa and BD FACS CantoII multicolor flow cytometers. Prior to each experiment, the CST program was run on both instruments to ensure proper calibration and optimal performance. Negative control, single stain, and fluorescence‐minus‐one (FMO) compensation tubes were prepared for multicolor analysis, with FMO controls employed to sequentially determine the negative and positive boundaries for each cell population. The voltage and fluorescence compensation settings for each channel were manually adjusted to ensure accurate representation of the target cell populations on the flow cytometric plots. Peripheral blood mononuclear cells (PBMCs) from different experimental groups were stained with the following surface markers: FVS‐BV421, CD45‐AF700 (BioLegend, 304024), CD3‐PerCP (BioLegend, 300326), CD4‐APC (BioLegend, 300514), CD8‐BV510 (BioLegend, 344732), CD56‐FITC (BioLegend, 318304), and TCRαβ‐BV605 (BD, 745088). Following surface staining, the cell membrane was permeabilized using the BD IntraSure kit (BD, 641776). Intracellular staining was then performed by incubating the cells with anti‐MT2A antibody (Affinity, DF6755) for 40 min, followed by washing with PBS. The cells were subsequently incubated with a preadsorbed Goat Anti‐Rabbit IgG H&L (PE) secondary antibody (Abcam, ab72465) for 30 min. Finally, the MT‐high T‐cell population was analyzed by flow cytometry to assess the distribution and abundance of the target cell subset. Data were acquired and analyzed using standard flow cytometric protocols.

### 4.11 Detection of Intracellular ROS

Intracellular reactive oxygen species (ROS) production was measured using the fluorescent probe 2', 7'‐dichlorodihydrofluorescein diacetate (DCFH‐DA) and flow cytometry. T cells were first activated with the eBioscience Cell Stimulation Kit (Thermo Fisher, 00‐4970‐03) according to the manufacturer's instructions. Following stimulation, 1 × 10^6^ cells were incubated with 10 µM DCFH‐DA at 37°C for 30 min to allow for probe uptake and cleavage by intracellular esterases. Dimethyl sulfoxide (DMSO) was used as a negative control. After incubation, cells were washed twice with PBS to remove excess probe. The level of ROS production was then assessed by flow cytometry, with DCF fluorescence serving as an indicator of intracellular ROS levels. Data were acquired and quantified based on the fluorescence intensity of DCF‐positive cells.

### 4.12 Statistical Analyses

For scRNA‐seq data, comparisons of cell distributions between two groups were performed using the unpaired two‐tailed Wilcoxon rank‐sum test. Gene expression or gene characteristics between two groups of cells were compared using the unpaired two‐tailed Student's *t*‐test. The paired two‐tailed Wilcoxon rank‐sum test was used to analyze the distribution of cells within paired cell types. All statistical analyses and data visualizations were carried out using R, and specific statistical tests applied are detailed in the figure legends. Statistical significance was defined as *p* < 0.05. For other experiments, data are presented as the mean ± standard deviation (SD) of at least three independent biological replicates. Statistical analyses were conducted using GraphPad Prism (version 9.5). Differences between two groups were assessed using the Mann–Whitney U test, while one‐way analysis of variance (ANOVA) was used for comparisons among multiple groups. Post‐hoc tests were applied where appropriate, and significance was defined as *p* < 0.05.

## Author Contributions

W.W., Y.C., C.C., and Y.C. designed the study. Z.L., J.L., and J.W. engaged in the acquisition, analysis, and interpretation of the data. X.R., M.M., H.L., Z.F., J.L., H.D., F.S., Y.L., and M.X. participated in specimen collection and data acquisition. M.Z., R.Z., Y.F., Z.W., Z.L., and Y.C. participated in flow cytometry and immunofluorescence data analysis. W.W., Y.C., C.C., Y.C., C.J.C., S.Y., and Z.L. drafted the article and all the authors approved the final manuscript.

## Conflicts of Interest

The authors declare no conflicts of interest.

## Supporting information




**Supporting File**: advs74350‐sup‐0001‐SuppMat.docx.

## Data Availability

The raw sequence data reported in this paper have been deposited in the Genome Sequence Archive (Genomics, Proteomics & Bioinformatics 2021) [46] in National Genomics Data Center (Nucleic Acids Res 2022) [47], China National Center for Bioinformation/Beijing Institute of Genomics, Chinese Academy of Sciences (GSA‐Human: HRA009896) and are publicly accessible at https://ngdc.cncb.ac.cn/gsa‐human. All requests for raw data will be promptly reviewed by the corresponding authors to determine whether the request is subject to any confidentiality obligations. Any data that can be shared will be released via a material transfer agreement. The published expression datasets analyzed in this work were deposited at Code Ocean (https://codeocean.com/capsule/8321305/tree/v1) and ArrayExpress (https://www.ebi.ac.uk/biostudies/arrayexpress), accession number: E‐MTAB‐8581.
