# Supplementary material for: Single‐Cell RNA Sequencing of Human Lung Tissues Reveals Metallothionein‐Positive T Cells as a Novel Potential Marker of Susceptibility to Chronic Obstructive Pulmonary Disease
Source: Adv Sci (Weinh). 2026 Feb 13;13(22):e09332. doi: 10.1002/advs.202509332 (PMC13088339; doi:10.1002/advs.202509332)
Supplement: Supplementary file 1 — Supporting File: advs74350‐sup‐0001‐SuppMat.docx. [file ADVS-13-e09332-s001.docx]

Supplementary Materials for

**Single-Cell RNA Sequencing of Human Lung Tissues Reveals Metallothionein-positive T cells as a Novel Potential Marker of Susceptibility to Chronic Obstructive Pulmonary Disease**

Zengqing Liu^1^, Xiaoxia Ren^2,3,4^, Mi Mu^5^, Huanyu Long^6^, Jialu Lv^2,4,1,3^, Zhihong Feng^7^, Jie Liu^1,2,3^, Jiajia Wang^8^, Hui Deng^8^, Fanyu Shi^1^, Muzhi Zhang^1^, Ruoyang Zhang^1^, Yang Liu^1^, Mengyu Xu^1^, Yang Fan^8^, Zengtao Wang^8^, Zhe Lv^1,3^, Yan Chen^1,3^, Chris J. Corrigan^9^, Sun Ying^1,3^, Yahong Chen^6*^, Chen Chen^8*^, Ye Cui^1,3*^, Wei Wang^1,2,3*^

Corresponding author: Ye Cui, Department of Immunology, School of Basic Medical Sciences, Capital Medical University, #10 Xi Tou Tiao, You An Men Wai, Feng Tai District, Beijing 100069, China. E-mail: [yecui@ccmu.edu.cn](mailto:yecui@ccmu.edu.cn)

Wei Wang, Department of Immunology, School of Basic Medical Sciences, Capital Medical University, #10 Xi Tou Tiao, You An Men Wai, Feng Tai District, Beijing 100069, China. E-mail: [wy_robin@ccmu.edu.cn](mailto:wy_robin@ccmu.edu.cn)

Chen Chen, Biomedical Innovation Center, Beijing Shijitan Hospital, Capital Medical University, Beijing 100038, China. E-mail: [chenchen1@ccmu.edu.cn](mailto:chenchen1@ccmu.edu.cn)

Yahong Chen, Department of Respiratory Medicine, Beijing Xuanwu Hospital, Capital Medical University, Beijing, China. E-mail: [chenyahong@vip.sina.com](mailto:chenyahong@vip.sina.com)

**The PDF file includes:**

Supplementary Text

Figs. S1 to S10

Tables S1 to S3

References

Fig. S1.


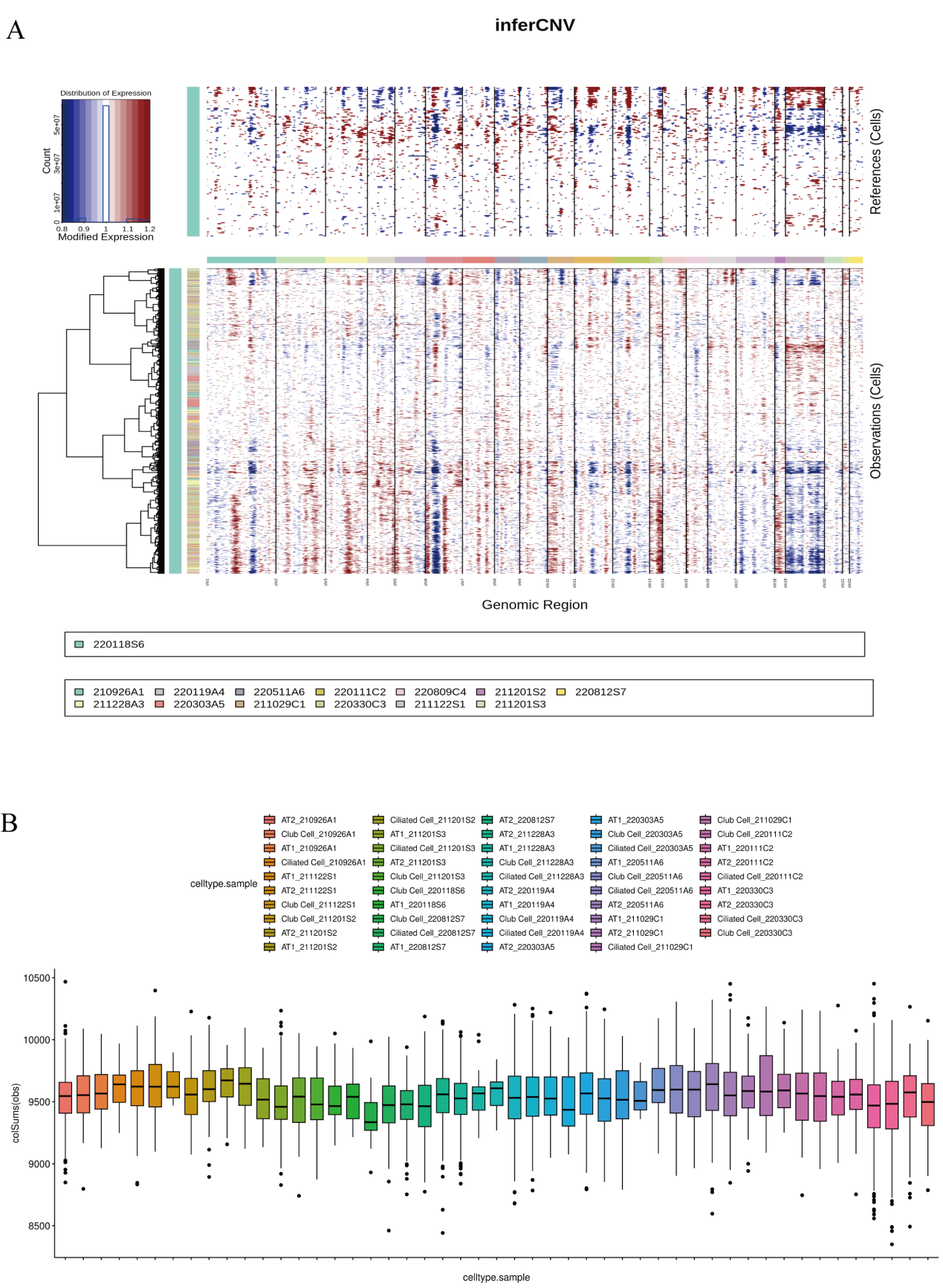


**Figure S1. Graded heat map and boxplot showing copy number variations(CNVs) of epithelial cells from lung tissues of all samples.** (A) Epithelial cells of 220926S6 are used as a control reference. Red: gains; blue: losses. (B) With normal cells (220926S6) as the reference, the changes in gene expression intensity at various genomic locations were analyzed across different groups of cells. A relative chromosome copy number between 0.9 and 1.1 is considered a normal copy event.

Fig. S2.


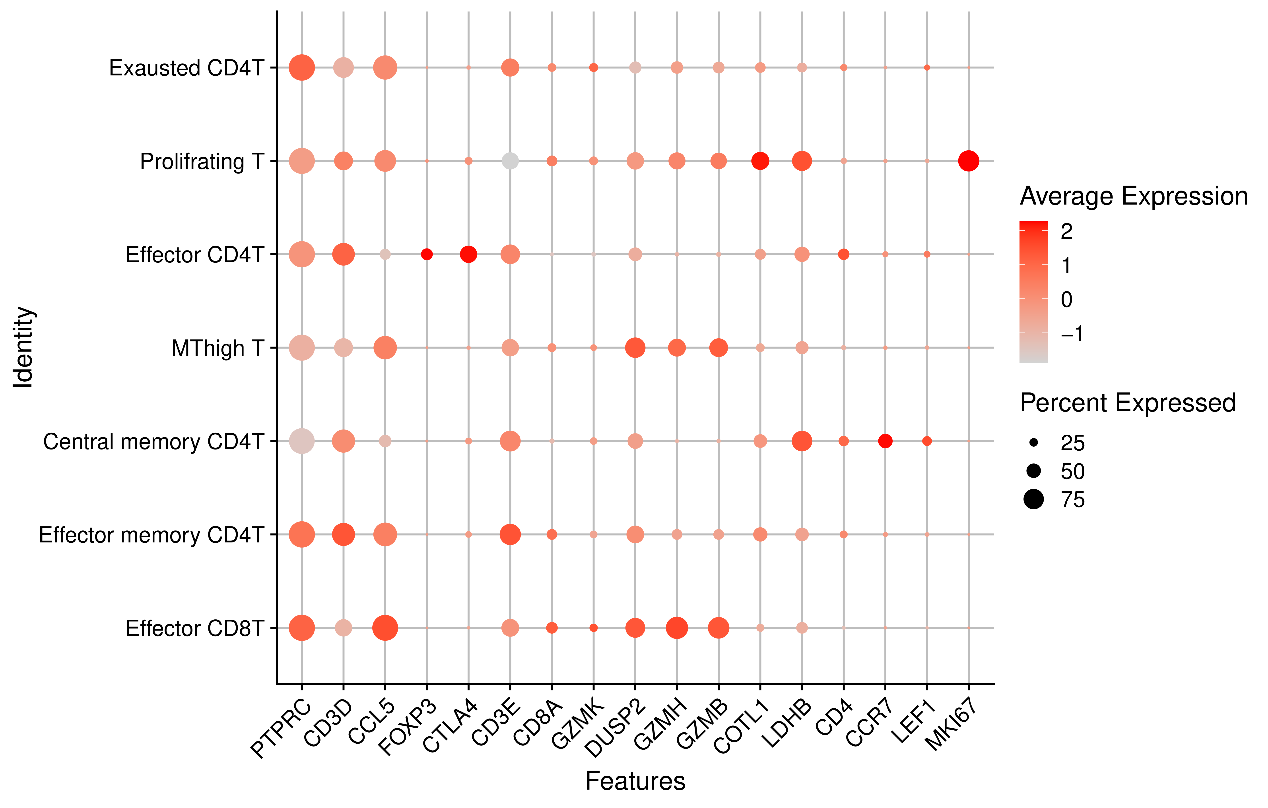


**Figure S2. Marker gene expression across identified T cell subtypes.** The dot plot shows the average expression level (color intensity) and the percentage of cells expressing each marker gene (dot size) for every T cell subtype.

Fig. S3


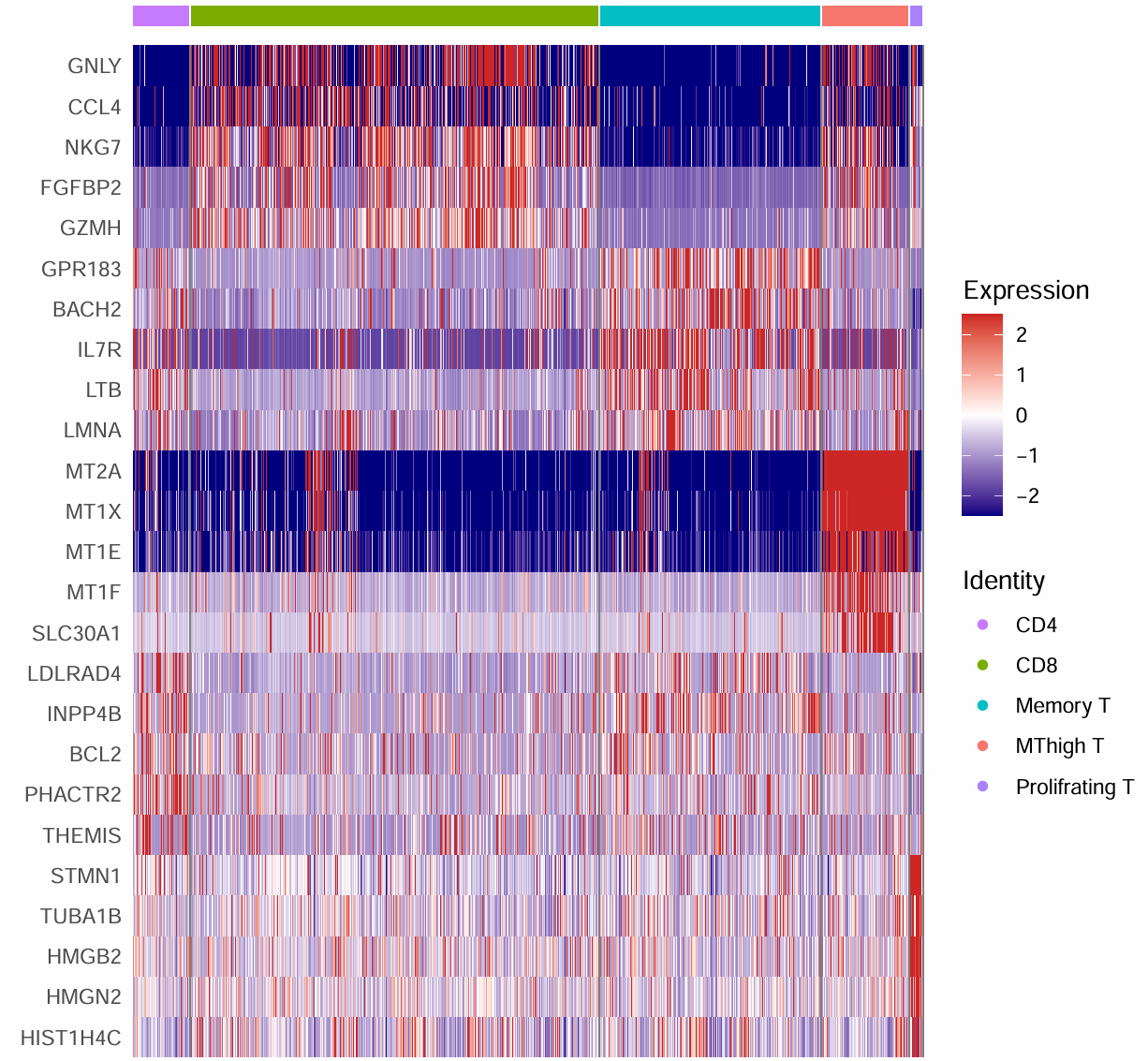


**Figure S3. MT-high T cells possess a distinct transcriptional signature that differentiates them from other T cell subsets.** The MT-high cluster is unequivocally defined by the massive and coordinate upregulation of metallothionein genes (e.g., *MT1E*, *MT1X*, *MT1F*, *MT2A*), establishing its unique molecular identity.

Fig. S4.


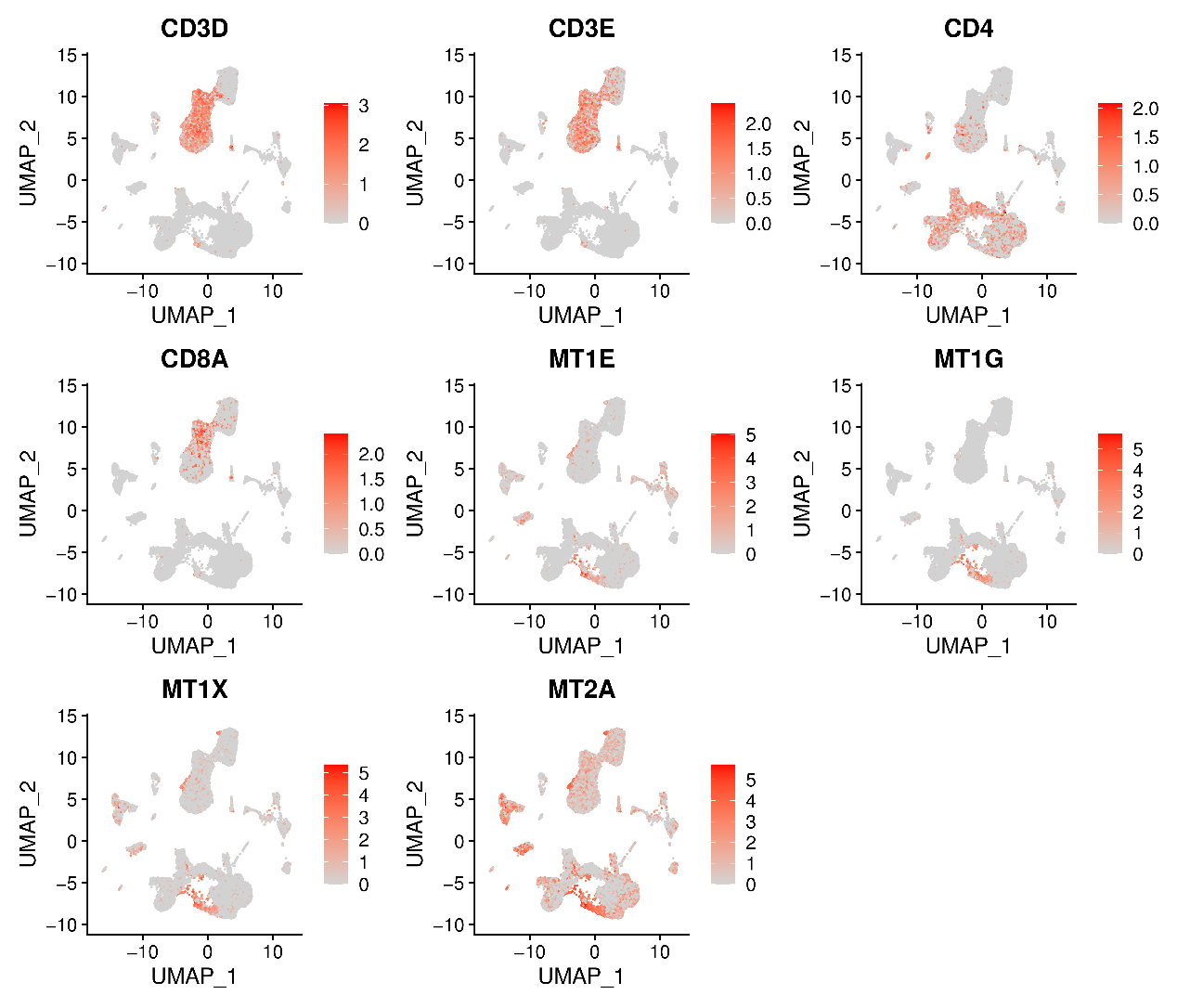


**Figure S4. Expression of metallothionein genes in public scRNA-seq datasets of normal human lung** (<https://codeocean.com/capsule/8321305/tree/v1>) (CD3D, CD3E: T cells; CD4: CD4^+^ T cells; CD8A: CD8^+^ T cells; MT1E, MT1G, MT1X, MT2A: metallothionein genes)

Fig. S5


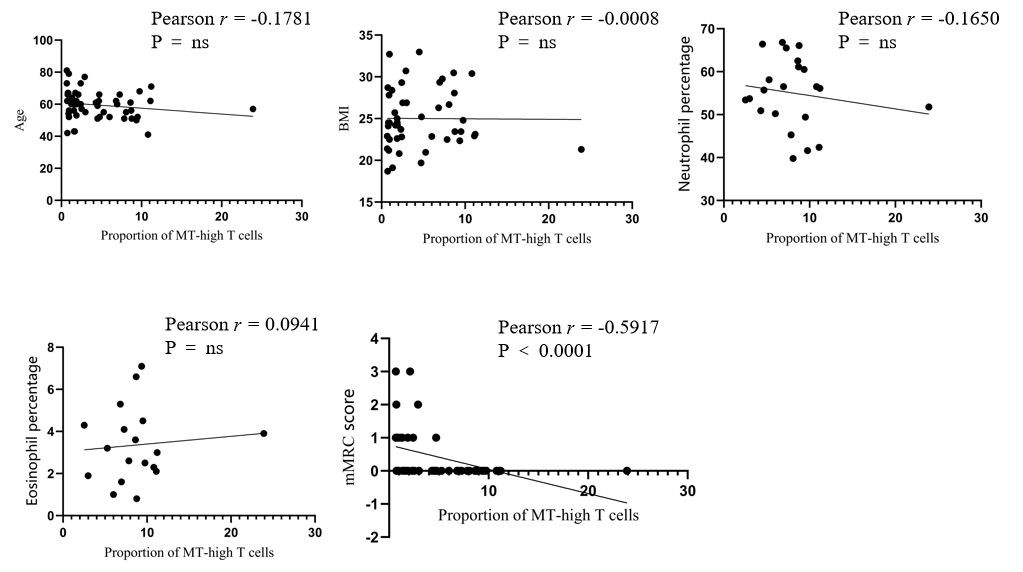


**Figure S5. MT-high T cell frequency correlates negatively with COPD symptom severity.** Pearson correlation analysis shows a significant negative association between the percentages of MT-high T cells and the mMRC score, but not with other demographic or inflammatory parameters.

Fig. S6.


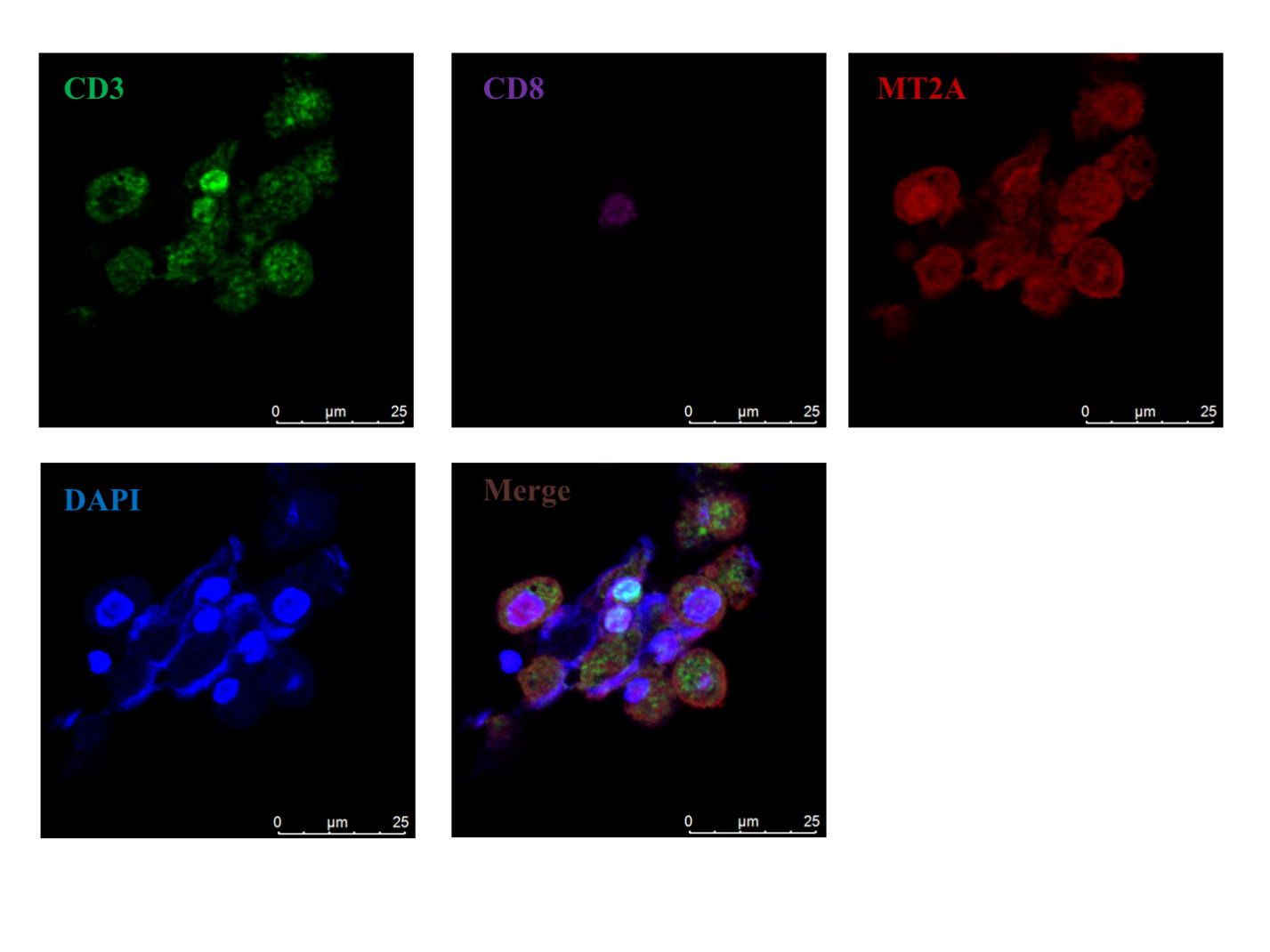


**Figure S6. Immunofluorescence staining of lung tissue sections from control subjects.** The figure shows the spatial proximity of CD8^+^ T cells and MT-high T cells, with CD3 labeled in green, CD8 in purple, MT2A in red, and nuclear staining in blue.Scale bars: 25μm.

Fig. S7.


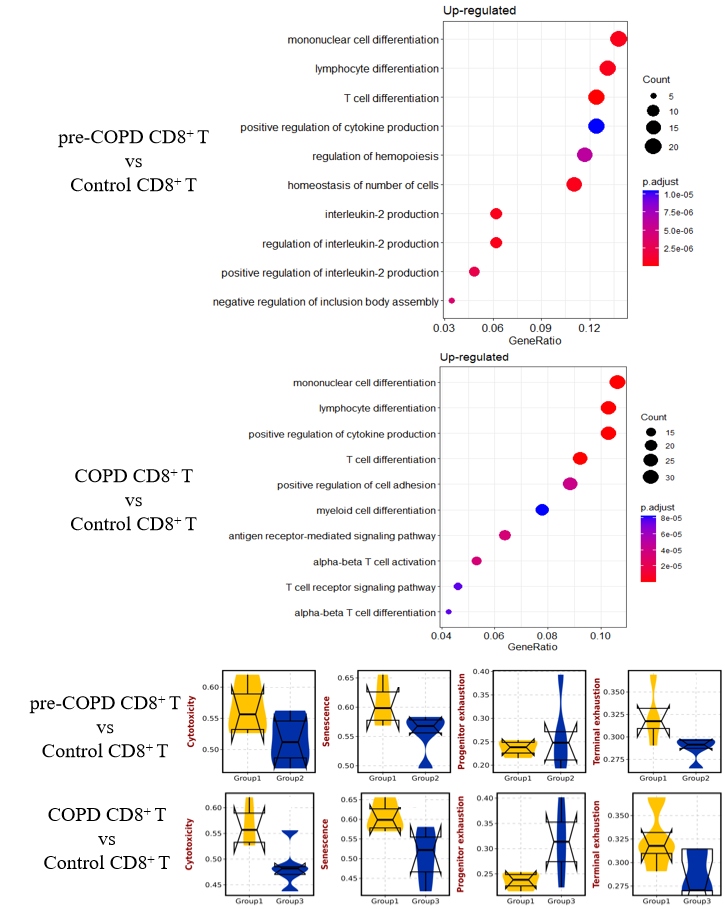


**Figure S7. Functional enrichment analysis of CD8^+^ T cells across different patient groups.**

Fig. S
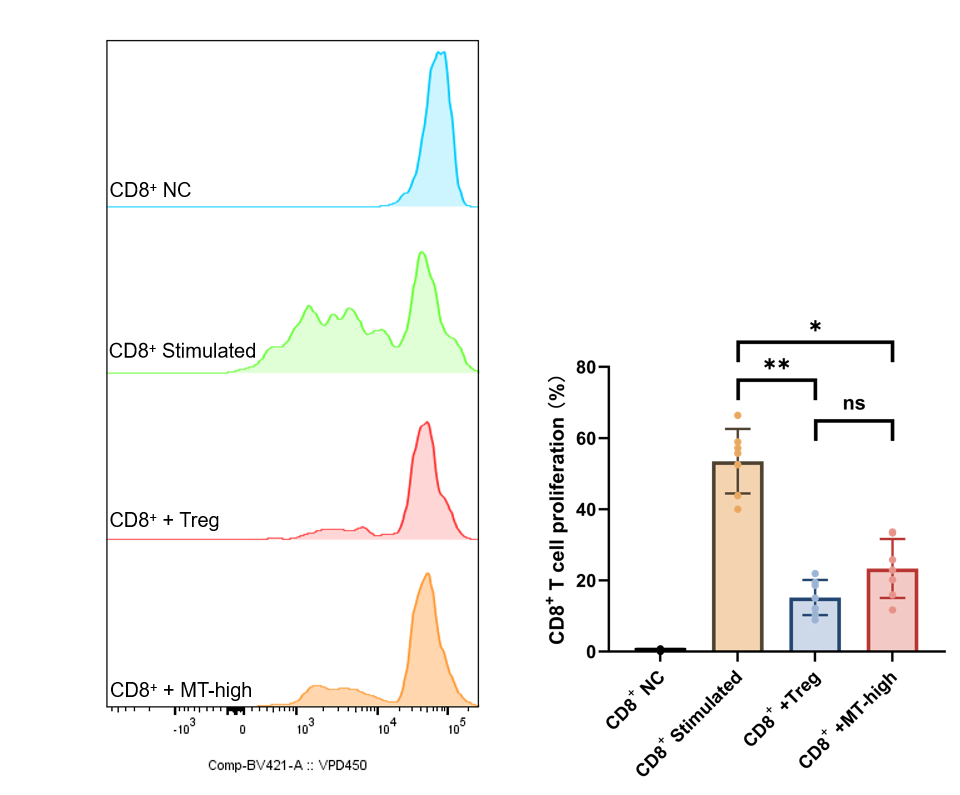
8

**Figure S8. Proliferation of CD8^+^ T cells is inhibited by co-culture with MT-high T cells. VPD450-labeled peripheral blood CD8^+^ T cells were stimulated with anti-CD3/CD28 antibodies.** Representative histograms show robust proliferation in the stimulated group, which was significantly suppressed by the addition of either Tregs (positive control) or MT-high T cells. Data represent the mean ± SD from seven donors; Data were analyzed using a one-way repeated measures ANOVA followed by Tukey's multiple comparisons test. NS, not significant; * *p* <0.05; ** *p*< 0.01.

Fig. S9


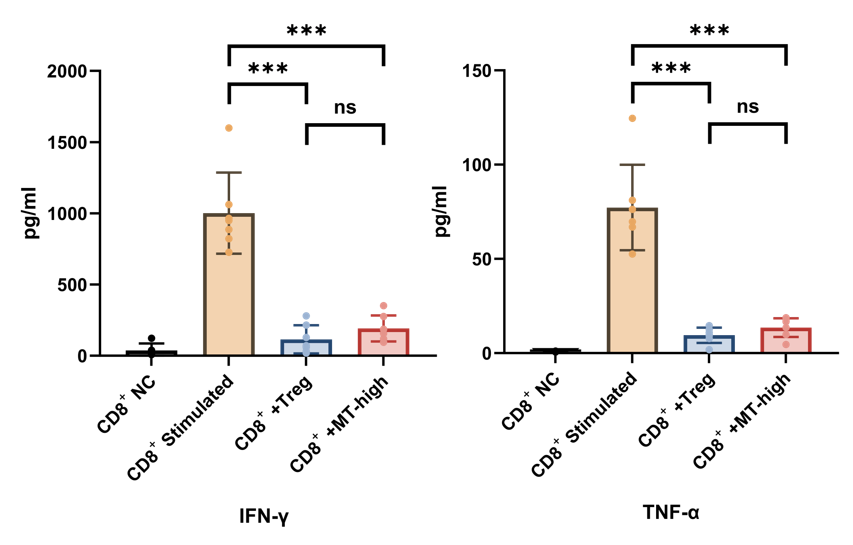


**Figure S9. MT-high T cells inhibit the secretion of pro-inflammatory cytokines by activated CD8^+^ T cells.** Concentrations of IFN-γ (left panel) and TNF-α (right panel) in culture supernatants were measured by ELISA. Co-culture with MT-high T cells significantly reduced the production of both cytokines from anti-CD3/CD28 stimulated CD8^+^ T cells. Data are presented as mean ± SD from seven independent donors. Statistical significance was determined by one-way ANOVA with Tukey's post-hoc test for multiple comparisons. NS, not significant; * *p* <0.05; *** *p*< 0.001.

Fig. S10.

**
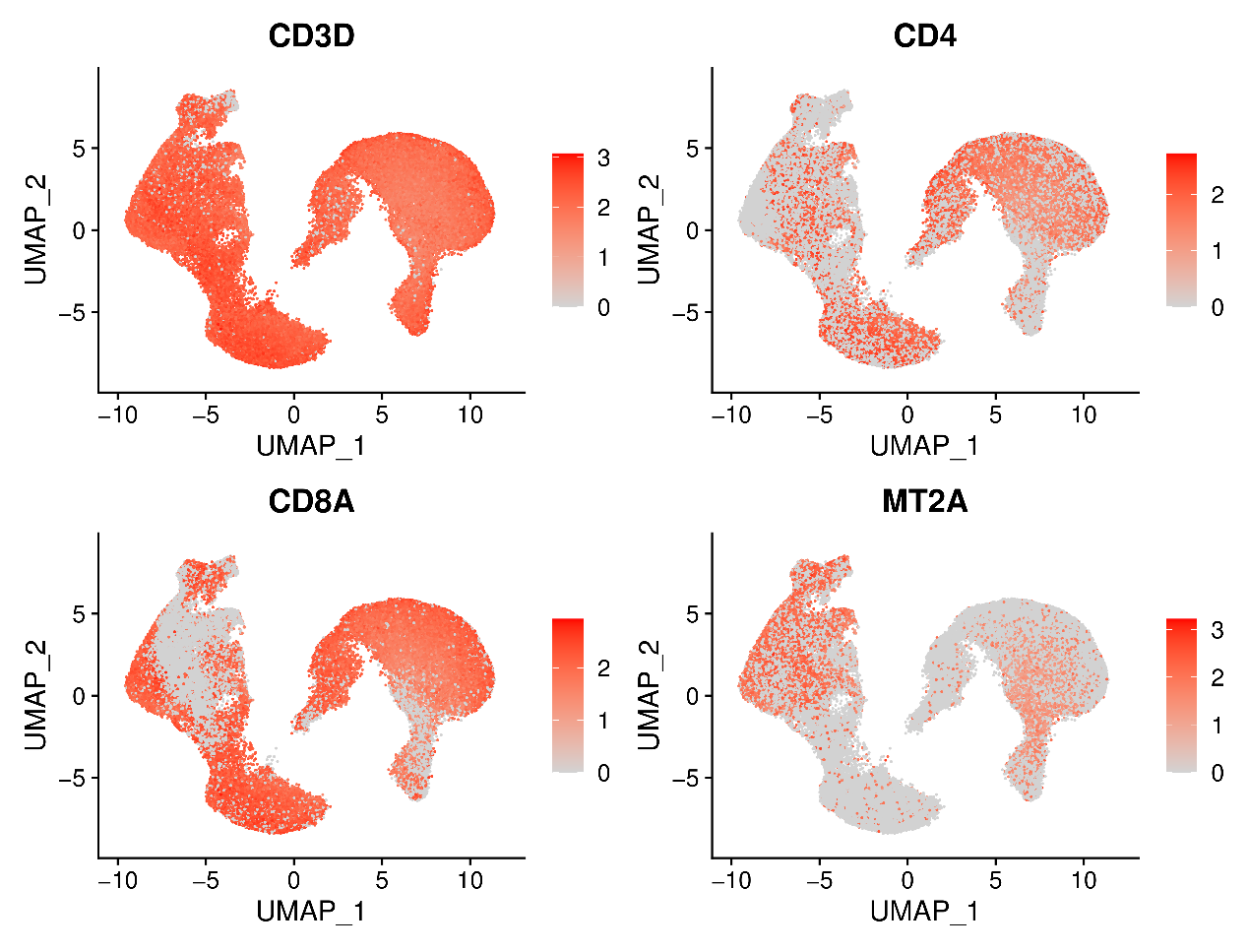
**

**Figure S10. Expression of metallothionein genes in public scRNA-seq datasets of normal human’s thymus** (data have been deposited to ArrayExpress, <https://www.ebi.ac.uk/biostudies/arrayexpress>, accession number: E-MTAB-8581).

Table S1.

| **Sample ID** | **Age** | **Gender** | **Diagnosis** | **History of smoking** | **Smoking**  **Exposure**  **(pack-years)** | **Comorbidity** |
| --- | --- | --- | --- | --- | --- | --- |
| 211029C1 | 39 | Female | Pulmonary tuberculosis | Never-smoker | 0 | - |
| 220111C2 | 49 | Female | Pulmonary nodule | Never-smoker | 0 | - |
| 220330C3 | 47 | Female | Pulmonary adenocarcinoma | Never-smoker | 0 | - |
| 220330C3 | 48 | Male | Pulmonary adenocarcinoma | Never-smoker | 0 | - |
| 210926A1 | 49 | Male | Pulmonary nodule | Current smoker | - | Diabetes mellitus |
| 211228A3 | 48 | Male | Pulmonary capillary hemangiomatosis | Former smoker | 15 | - |
| 220119A4 | 41 | Male | Minimally invasive adenocarcinoma of the lung | Current smoker | 10 | Hypertension, diabetes mellitus |
| 220303A5 | 40 | Male | Invasive adenocarcinoma of the lung | Former smoker | 10 | Coronary heart disease, hyperglycemia |
| 220511A6 | 40 | Male | Squamous-cell carcinoma of the lung | Former smoker | 10 | Bronchiectasis |
| 211122S1 | 63 | Male | COPD, Invasive adenocarcinoma of the lung | Never-smoker | 0 | Hypertension |
| 211201S2 | 57 | Male | COPD, Invasive adenocarcinoma of the lung | Current smoker | 2.5 | - |
| 211201S3 | 74 | Male | COPD, Squamous-cell carcinoma of the lung | Former smoker | 40 | Coronary heart disease |
| 220118S6 | 60 | Male | COPD, emphysema | Current smoker | 60 | - |
| 220812S7 | 64 | Male | COPD, Small cell lung cancer | Current smoker | 40 | Diabetes mellitus, coronary heart disease |

**Table S1. Clinical information of patients for single-cell RNA sequencing**

Table S2.

| **Sample ID** | **Gender** | **Age** | **History of smoking** | **Smoking**  **Exposure (pack-years)** | **FEV1/FVC%** |
| --- | --- | --- | --- | --- | --- |
| NC1 | Female | 51 | Never-smoker | 0 | 80 |
| NC2 | Female | 52 | Never-smoker | 0 | 83 |
| NC3 | Male | 62 | Never-smoker | 0 | 81 |
| NC4 | Male | 61 | Never-smoker | 0 | 80 |
| NC5 | Male | 60 | Never-smoker | 0 | 76 |
| NC6 | Male | 55 | Never-smoker | 0 | 73 |
| NC7 | Male | 57 | Never-smoker | 0 | 94 |
| NC8 | Male | 50 | Never-smoker | 0 | 72 |
| NC9 | Male | 57 | Never-smoker | 0 | 73 |
| NC10 | Male | 52 | Never-smoker | 0 | 70 |
| NC11 | Male | 60 | Never-smoker | 0 | 75 |
| NC12 | Female | 56 | Never-smoker | 0 | 86 |
| NC13 | Male | 67 | Never-smoker | 0 | 80 |
| NC14 | Male | 57 | Never-smoker | 0 | 86 |
| NC15 | Female | 54 | Never-smoker | 0 | 75 |
| NC16 | Male | 52 | Never-smoker | 0 | 80 |
| NC17 | Male | 59 | Never-smoker | 0 | 70 |
| CS1 | Male | 65 | Current smoker | 80 | 70 |
| CS2 | Male | 71 | Current smoker | 80 | 77 |
| CS3 | Male | 41 | Current smoker | 50 | 83 |
| CS4 | Male | 68 | Current smoker | 60 | 75 |
| CS5 | Male | 52 | Current smoker | 60 | 87 |
| CS6 | Male | 66 | Current smoker | 50 | 72 |
| CS7 | Male | 51 | Current smoker | 50 | 71 |
| CS8 | Male | 56 | Current smoker | 80 | 70 |
| CS9 | Male | 62 | Current smoker | 80 | 72 |
| CS10 | Male | 55 | Current smoker | 50 | 70 |
| CS11 | Male | 55 | Current smoker | 50 | 70 |
| CS12 | Male | 62 | Current smoker | 80 | 95 |
| CS13 | Male | 61 | Current smoker | 50 | 77 |
| CS14 | Male | 51 | Current smoker | 80 | 75 |
| pre-COPD-1 | Male | 52 | Current smoker | 80 | 70.68 |
| pre-COPD-2 | Male | 59 | Current smoker | 60 | 76.01 |
| pre-COPD-3 | Female | 61 | Never-smoker | 0 | 72.83 |
| pre-COPD-4 | Male | 54 | Former smoker | 10 | 70.09 |
| pre-COPD-5 | Female | 60 | Never-smoker | 0 | 71.84 |
| pre-COPD-6 | Male | 66 | Never-smoker | 0 | 71.32 |
| pre-COPD-7 | Male | 52 | Never-smoker | 0 | 70.82 |
| pre-COPD-8 | Male |  | Never-smoker | 0 | 72 |
| pre-COPD-9 | Male | 60 | Never-smoker | 0 | 72.46 |
| pre-COPD-10 | Male | 60 | Never-smoker | 0 | 73.39 |
| pre-COPD-11 | Female | 64 | Never-smoker | 0 | 73.12 |
| pre-COPD-12 | Male | 73 | Current smoker | 60 | 72 |
| pre-COPD-13 | Male | 62 | Former smoker | 0 | 73 |
| COPD-1 | Male | 60 | Current smoker | 60 | 68 |
| COPD-2 | Male | 81 | Former smoker | 60 | 54 |
| COPD-3 | Male | 79 | Never-smoker | 0 | 68.94 |
| COPD-4 | Male | 73 | Former smoker | 60 | 26.15 |
| COPD-5 | Male | 66 | Former smoker | 80 | 67.34 |
| COPD-6 | Male | 62 | Current smoker | 80 | 31.36 |
| COPD-7 | Male | 67 | Current smoker | 80 | 42.5 |
| COPD-8 | Male | 42 | Former smoker | 80 | 56.42 |
| COPD-9 | Male | 56 | Current smoker | 80 | 48.79 |
| COPD-10 | Male | 66 | Former smoker | 50 | 62.83 |
| COPD-11 | Female | 62 | Never-smoker | 0 | 57.97 |
| COPD-12 | Male | 73 | Former smoker | 80 | 60.00 |
| COPD-13 | Female | 60 | Never-smoker | 0 | 62.4 |
| COPD-14 | Female | 53 | Never-smoker | 0 | 66.54 |
| COPD-15 | Male | 67 | Former smoker | 80 | 67.00 |
| COPD-16 | Male | 77 | Former smoker | 80 | 58.62 |
| COPD-17 | Male | 43 | Never-smoker | 0 | 62 |
| COPD-18 | Male | 69 | Current smoker | 50 | 61.39 |
| COPD-19 | Male | 43 | Never-smoker | 0 | 63.62 |
| COPD-20 | Male | 67 | Current smoker | 80 | 49.71 |
| COPD-21 | Male | 56 | Current smoker | 80 | 63.83 |
| COPD-22 | Male | 64 | Former smoker | 40 | 59.04 |
| COPD-23 | Male | 67 | Never-smoker | 0 | 54 |
| COPD-24 | Male | 69 | Former smoker | 80 | 68 |
| COPD-25 | Male | 61 | Current smoker | 80 | 61.9 |
| COPD-26 | Male | 55 | Never-smoker | 0 | 63 |
| COPD-27 | Male | 67 | Former smoker | 40 | 67.6 |
| COPD-28 | Male | 35 | Never-smoker | 0 | 65 |
| COPD-29 | Male | 77 | Never-smoker | 0 | 69 |
| COPD-30 | Male | 72 | Former smoker | 80 | 65 |
| COPD-31 | Male | 75 | Current smoker | 50 | 67 |
| COPD-32 | Male | 75 | Current smoker | 40 | 67.3 |
| COPD-33 | Male | 67 | Never-smoker | 0 | 60 |
| COPD-34 | Male | 74 | Former smoker | 80 | 49 |
| COPD-35 | Male | 59 | Former smoker | 80 | 51.1 |
| COPD-36 | Male | 53 | Former smoker | 80 | 57.3 |

**Table S2. Clinical information of the peripheral blood collection cohort.**

Table S3.

| **Group** | **percentage of MT-high**  **CD3^+^ T cells (%)** | **percentage of MT-high**  **DN T cells (%)** |
| --- | --- | --- |
| NC | 8.56 | 10.69 |
| CS | 7.54 | 8.48 |
| pre-COPD | 2.45 | 2.50 |
| COPD | 1.36 | 1.65 |

**Table S3. Quantitative Flow Cytometric Analysis of MT-high T Cells in Peripheral Blood**
